# Natural saponins and macrophage polarization: Mechanistic insights and therapeutic perspectives in disease management

**DOI:** 10.3389/fphar.2025.1584035

**Published:** 2025-05-09

**Authors:** Beibei Xiong, Huan Wang, Yi-Xuan Song, Wen-Ying Lan, Jiangtao Li, Fang Wang

**Affiliations:** ^1^ Department of Oncology, The First People’s Hospital of Shuangliu District, Chengdu, China; ^2^ School of Pharmacy, Chengdu University of Traditional Chinese Medicine, Chengdu, China; ^3^ Chengdu First People’s Hospital, Chengdu, China

**Keywords:** saponin, macrophage polarization, molecular mechanism, pharmacokinetics, bioavailability, natural products

## Abstract

Macrophage polarization plays a pivotal role in immune homeostasis and disease progression across inflammatory, neoplastic, and metabolic disorders. Saponins, which are natural compounds with steroidal/triterpenoid structures, demonstrate therapeutic potential through immunomodulatory, anti-inflammatory, and anti-tumor activities. This study aims to highlight the potential of key saponins—such as ginsenosides, astragaloside IV, dioscin, platycodin D, pulsatilla saponins, and panax notoginseng saponins—in modulating macrophage polarization and enhancing conventional therapies, particularly in oncology. We conducted structured searches in PubMed, Google Scholar, and SciFinder (2013–2024) using controlled vocabulary, including “saponins,” “macrophage polarization,” and “therapeutic effects.” Our findings demonstrate that saponins significantly modulate immune responses and improve treatment efficacy. However, clinical translation is hindered by challenges such as poor bioavailability and safety concerns, which limit systemic exposure and therapeutic utility. To overcome these barriers, innovative delivery strategies, including nanoemulsions and engineered exosomes, are essential for enhancing pharmacokinetics and therapeutic index. Future research should prioritize elucidating the molecular mechanisms underlying saponin-mediated macrophage polarization, identifying novel therapeutic targets, and optimizing drug formulations. Addressing these challenges will enable the restoration of immune balance and more effective management of diverse diseases.

## Graphical Abstract

**Figure:**
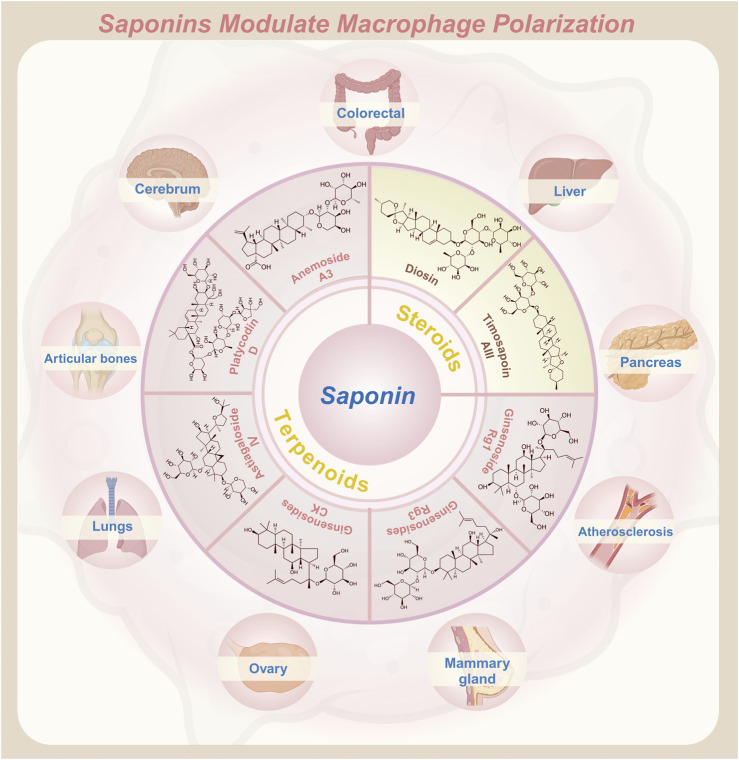
Saponins modulate the polarization state of macrophages, enhancing their therapeutic potential in various diseases.

## 1 Introduction

Macrophages are versatile immune cells essential for host defense and tissue homeostasis, performing critical functions including pathogen clearance, removal of damaged cells, and immune regulation through cytokine release (Li et al., 2018; Chen et al., 2023). Their functional plasticity stems from polarization—a dynamic process where they adopt distinct phenotypes in response to environmental signals. Traditionally classified as pro-inflammatory M1 or anti-inflammatory M2 subtypes, macrophage phenotypes actually exist on a dynamic spectrum. M1 macrophages drive pathogen clearance and inflammation, while M2 macrophages promote tissue repair and inflammation resolution. However, in tumors, M2-like macrophages undergo metabolic reprogramming. For example, S100A4^+^ tumor-associated macrophages (TAMs) upregulate fatty acid oxidation (FAO) and CD36-mediated lipid uptake via PPAR-γ-dependent pathways (Liu et al., 2021). This not only reinforces M2 polarization but also promotes tumor progression by suppressing anti-tumor immunity. Meanwhile, balancing these phenotypes is crucial for maintaining immune equilibrium and greatly influences disease progression (Wang et al., 2024). A paradigm of this balance is evident in atherosclerosis. During early plaque development, pro-inflammatory M1 macrophages dominate the lesion, secreting cytokines such as TNF-α and IL-6 to recruit monocytes and amplify endothelial inflammation, thereby accelerating lipid deposition (Shioi and Ikari, 2018). However, persistent M1 activity destabilizes plaques via matrix metalloproteinases (MMPs), whereas M2 macrophages stabilize plaques through IL-10, TGF-β, and collagen synthesis. Clinically, vulnerable plaques show a 3-fold higher M1/M2 ratio (CD68^+^/CD163^+^) than stable lesions, directly correlating with acute coronary syndrome risk (Yang et al., 2019a). Therapeutic strategies targeting macrophage polarization are emerging. Several FDA-approved drugs have shown significant promise in modulating M2 macrophage polarization and expansion, offering new avenues for therapeutic intervention. For instance, rapamycin effectively regulates M2 macrophage polarization (He et al., 2021), while Tofacitinib (Xeljanz^®^) targets their expansion (Flanagan et al., 2014), both demonstrating notable clinical efficacy in managing conditions such as organ transplant rejection and rheumatoid arthritis. In parallel, a CSF1R inhibitor is also in clinical trials for cancer and chronic inflammation, aiming to reprogram the tumor microenvironment (TME) (Wen et al., 2023). Collectively, these advancements underscore the translational potential of macrophage polarization research across diseases.

Building on these successful applications, recent studies highlight the potential of natural bioactive compounds in regulating macrophage polarization, with saponins emerging as a particularly prominent class of bioactive substances (Joyce et al., 2021). Saponins are structurally characterized by a sugar moiety linked to an aglycone (steroidal or triterpenoid backbone) via a glycosidic bond. They are broadly categorized into triterpenoid and steroidal saponins based on the nature of their aglycones (Timilsena et al., 2023). Triterpenoid saponins, exemplified by dioscin, exhibit potent anti-inflammatory, antioxidant, and antitumor activities, while steroidal saponins such as ginsenosides similarly demonstrate significant pharmacological effects (Augustin et al., 2011; Passos et al., 2022b). Increasing evidence highlights the ability of plant-derived saponins, including ginsenosides and astragalosides, to regulate macrophage polarization, making them promising candidates for immune modulation (Jolly et al., 2024). These compounds exert their immunomodulatory effects through specific signaling pathways. For instance, ginsenosides have been shown to activate the STAT6 pathway, driving macrophages toward the M2 phenotype, thereby enhancing anti-inflammatory responses and supporting tissue repair (Chen et al., 2023).

Despite the substantial progress in identifying the immunomodulatory roles of saponins, a comprehensive understanding of their therapeutic potential in macrophage polarization remains elusive. This review addresses this knowledge gap by systematically examining recent advances in the field, with a focus on signal transduction pathways, pharmacokinetics, and safety profiles associated with saponins. Additionally, this work explores the prospects of combination therapies involving saponin compounds and identifies key research priorities to facilitate further advancement. By integrating current knowledge, our review aims to provide a robust scientific foundation for the development of novel therapeutic strategies targeting macrophage polarization and immune modulation.

## 2 The molecular mechanism of macrophage polarization

Macrophages, central to the innate immune system, display exceptional plasticity, allowing adaptation to tissue-specific demands and inflammatory cues. This flexibility drives macrophage polarization, a dynamic shift between phenotypes regulated by cytokines, growth factors, and microbial signals that influence gene expression via distinct intracellular pathways. Before examining the molecular mechanisms and signaling pathways involved in macrophage polarization, it is essential to first understand its classification framework.

### 2.1 Classification of macrophage polarization

**FIGURE 1:**
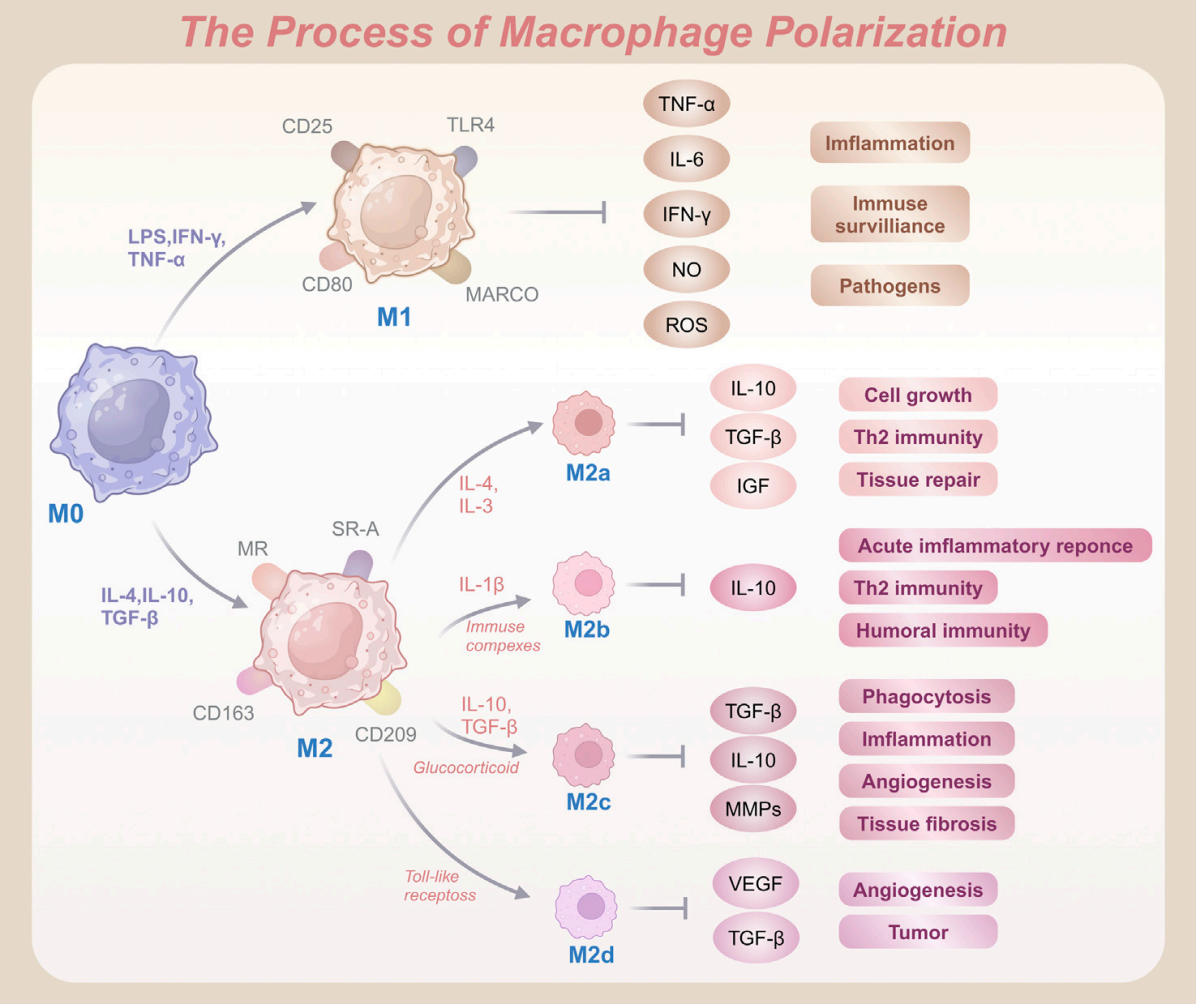
Schematic representation of macrophage polarization from M0 to pro-inflammatory M1 and anti-inflammatory M2 phenotypes, highlighting key activation markers and functional outcomes.

To understand the functional heterogeneity of macrophages, polarization is typically classified into two main phenotypes: M1 (classically activated macrophages) and M2 (alternatively activated macrophages) (Figure 1). M1 macrophages are characterized by surface markers such as MHC II, CD40, CD80, and CD86 (Martin and García, 2021). Under stimulation with lipopolysaccharide (LPS), interferon-gamma (IFN-γ), or tumor necrosis factor-alpha (TNF-α), resting macrophages (M0) differentiate into pro-inflammatory M1 macrophages (Wang et al., 2018). These macrophages express receptors including TLR4, macrophage receptor with collagenous structure (MARCO), CD25, and CD80 (Zhang et al., 2018), and secrete high levels of pro-inflammatory cytokines such as TNF-α, interleukin-6 (IL-6), IFN-γ, nitric oxide (NO), and reactive oxygen species (ROS) (Zheng et al., 2020). Acting as drivers of inflammation, M1 macrophages enhance immune surveillance and directly kill pathogens (Collins et al., 2021). They also exhibit strong cytotoxic effects through the production of ROS and reactive nitrogen species (RNS), contributing to tissue damage and pathogenesis (Evans et al., 1997). Although these pro-inflammatory properties are vital for eliminating pathogens and activating host immunity, excessive recruitment of M1 macrophages can aggravate inflammatory damage to normal tissues. When this occurs, the repair-oriented functions of M2 macrophages become essential for restoring tissue homeostasis.

Unlike their M1 counterparts, M2 macrophages are associated with anti-inflammatory and regenerative functions. Common markers of M2 macrophages include CD163, CD204, and CD206 (Al-Matari et al., 2024). Upon stimulation by anti-inflammatory cytokines such as interleukin-4 (IL-4), interleukin-10 (IL-10), IL-13, or transforming growth factor-beta (TGF-β), macrophages polarize into the M2 phenotype (Zhang and Jagannath, 2025). Recognized by receptors such as the mannose receptor (MR), scavenger receptor A (SR-A), CD163, and CD209, and secrete bioactive molecules including resistin-like Fizz1, arginase-1 (Arg1), chitinase-3-like-protein-1 (CHI3L1), IL-10, and MR C-type 1 (Mrc1, also known as CD206) (van der Zande et al., 2021). These molecules play critical roles in tissue repair, wound healing, and immune modulation by reducing inflammation and promoting regeneration of damaged tissues (Wynn and Vannella, 2016). Additionally, M2 macrophages produce growth factors such as hepatocyte growth factor (HGF) and insulin-like growth factor 1 (IGF-1), which enhance tissue repair and minimize excessive immune reactions.

The M2 phenotype exhibits notable diversity across inflammatory and pathological conditions, unlike the relatively uniform M1 population. It is further subdivided into M2a, M2b, M2c, and M2d subtypes (Bhattacharya and Aggarwal, 2019). M2a macrophages, induced by IL-4 or IL-13, promote cell proliferation, tissue repair, and Th2-mediated immune responses by expressing markers such as MHC II, MR, and Arg1, and secreting cytokines such as IL-10, TGF-β, IGF, and fibronectin (Arora et al., 2018). M2b macrophages arise upon stimulation with interleukin-1β (IL-1β) or immune complexes. These cells express surface molecules such as MHC II and CD86, release IL-10, and suppress acute inflammation, promote humoral immunity, and drive Th2 responses (Wang et al., 2019b). M2c macrophages, often referred to as deactivated macrophages, are associated with immune suppression, angiogenesis, and tissue remodeling. Induced by IL-10, TGF-β, or glucocorticoids, they secrete high levels of TGF-β, IL-10, and matrix metalloproteinases (MMPs), facilitating tissue repair and fibrosis (Lurier et al., 2017). Furthermore, M2d macrophages, often termed TAMs, are activated by Toll-like receptor agonists and secrete vascular endothelial growth factors (VEGF) and IL-10. Critically, these macrophages drive angiogenesis and tumor progression by producing VEGF and TGF-β (Barilo et al., 2025). Collectively, these M2 subtypes highlight the functional adaptability of macrophages in diverse environments and pathologies.

### 2.2 Signaling pathways and regulatory mechanisms of macrophage polarization

**FIGURE 2:**
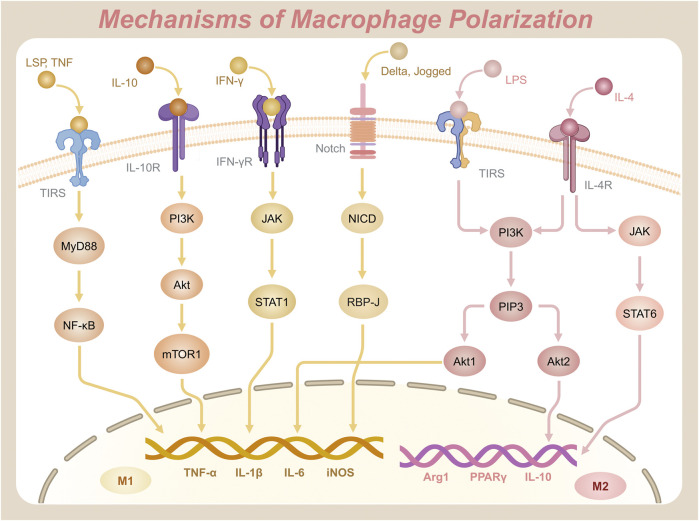
Key signaling pathways and molecular mechanisms driving the polarization of macrophages into M1 and M2 phenotypes.

Macrophages exhibit remarkable plasticity, transitioning from a quiescent state (M0) to distinct functional phenotypes—such as the pro-inflammatory M1 or the anti-inflammatory M2 type—in response to environmental cues. The molecular signaling pathways governing this polarization process are critical for understanding immune regulation and designing targeted therapies. Prominent pathways include the NF-κB, JAK/STAT, PI3K/Akt, and Notch signaling cascades, which operate in a highly coordinated manner to influence macrophage phenotypic outcomes (Figure 2).

#### 2.2.1 NF-κB signaling pathway

The NF-κB signaling pathway is a central regulatory axis in macrophages that governs innate immunity and inflammatory responses (Dorrington and Fraser, 2019). This pathway is mediated by the NF-κB family of transcription factors, comprising five members: NF-κB1 (p50), NF-κB2 (p52), RelA (p65), RelB, and c-Rel (Reale et al., 2018). The canonical pathway predominantly involves the p65/p50 heterodimer, whereas the RelB/p52 complex functions in the alternative NF-κB pathway (Ji et al., 2020). Notably, the non-canonical pathway relies on NF-κB-inducing kinase (NIK)-mediated phosphorylation of IKKα, which processes p100 to p52, enabling RelB/p52 nuclear translocation and activation of genes involved in lymphoid organogenesis and chronic inflammation (Pflug and Sitcheran, 2020). NF-κB proteins share a conserved Rel homology domain (RHD) at their N-terminal region, which facilitates DNA binding, dimerization, nuclear localization, and interaction with inhibitors of κB (IκB) (Hellweg, 2015). Post-translational modifications further regulate NF-κB activity: phosphorylation of p65 at Ser536 (by IKKβ or TBK1) enhances transcriptional activation, while K63-linked ubiquitination of IKKγ (NEMO) is essential for signalosome assembly (Sun et al., 2024). In the resting state, NF-κB dimers are sequestered in the cytoplasm by IκB, maintaining an inhibitory state (Mitchell and Carmody, 2018). Upon activation by inflammatory stimuli, such as pathogen-associated molecular patterns (PAMPs) binding to Toll-like receptors (TLRs), the IκB kinase (IKK) complex—comprising IKKα, IKKβ, and NEMO (IKKγ)—phosphorylates IκB, marking it for ubiquitination and subsequent proteasomal degradation. This degradation liberates NF-κB, allowing it to translocate to the nucleus, where it initiates transcription of genes encoding pro-inflammatory mediators, such as TNF-α, IL-1β, IL-6, and inducible nitric oxide synthase (iNOS), driving M1 polarization (Pflug and Sitcheran, 2020). Epigenetic regulation via histone acetyltransferases (p300/CBP) and deacetylases (HDAC3) modulates chromatin accessibility at NF-κB target loci, thereby fine-tuning cytokine production during macrophage polarization (Chakraborty et al., 2022). The adaptor protein Myeloid differentiation primary response 88 (MyD88) facilitates this activation by linking TLRs to downstream IKK complexes (Yu et al., 2020). Importantly, NF-κB crosstalks with other polarization pathways: STAT1 synergizes with NF-κB to amplify M1-associated genes, while PPARγ antagonizes NF-κB by recruiting co-repressors (NCoR/HDAC3) to p65, promoting M2 polarization and inflammation resolution (Oeckinghaus et al., 2011). In addition to its role in inflammatory cytokine production, NF-κB activation through the MyD88-dependent pathway coordinates with MAPK signaling to regulate inflammatory responses. Specifically, p38 MAPK modulates both the phosphorylation status of Stat1 and the transcriptional activity of NF-κB, while JNK influences the Jak1/Stat1 signaling axis (Ramsauer et al., 2002). This intricate crosstalk is evidenced by Stat1-mediated negative regulation of MyD88-dependent MMP-9 expression, creating a feedback mechanism that balances proinflammatory gene expression with anti-inflammatory modulation.

#### 2.2.2 JAK/STAT signaling pathway

The Janus kinase/signal transducer and activator of transcription (JAK/STAT) signaling pathway integrates cytokine-driven signals to mediate macrophage polarization. This pathway involves four JAK isoforms (JAK1, JAK2, JAK3, and Tyk2) and seven STAT proteins (STAT1, STAT2, STAT3, STAT4, STAT5a, STAT5b, and STAT6) (Starr and Hilton, 1999). JAK kinases are characterized by a kinase domain (JH1) and a pseudokinase domain (JH2). The JH2 domain regulates basal activity and prevents spontaneous activation, ensuring tight control of signaling initiation (Saharinen and Silvennoinen, 2002). In M1 polarization, IFN-γ binds to its receptor, activating JAK kinases, which phosphorylate STAT1. Phosphorylation of STAT1 at Tyr701 induces dimerization, while Ser727 phosphorylation (mediated by MAPK or mTOR pathways) enhances transcriptional activity, enabling robust expression of M1 markers like CXCL9 and iNOS (Barnholt et al., 2009). Phosphorylated STAT1 forms dimers that translocate to the nucleus, activating transcription of M1-specific genes and amplifying pro-inflammatory responses (Pilz et al., 2003). Conversely, M2 polarization relies on IL-4, which binds to the IL-4Rα receptor to activate JAK kinases, leading to the phosphorylation of STAT6 (He et al., 2020). STAT6 dimers translocate to the nucleus and interact with transcription factors such as KLF4 and PPARγ, promoting anti-inflammatory gene expression, such as Arg1, IL-10, and PPARγ, and driving M2 macrophage functions (Kapoor et al., 2015). Notably, STAT6 also recruits histone acetyltransferase p300 to the promoters of M2-associated genes, facilitating chromatin remodeling and sustained transcriptional activation (Shankaranarayanan et al., 2001). Negative regulation of this pathway is mediated by suppressor of cytokine signaling (SOCS) proteins. SOCS3 limits excessive STAT1 activation, while SOCS1/3 modulates M2 polarization dynamics. SOCS3 binds directly to phosphorylated JAK2 via its SH2 domain, which promotes ubiquitination and proteasomal degradation of the receptor complex. This mechanism terminates STAT1 signaling (Yu et al., 2015a). Dysregulation of SOCS proteins, especially insufficient SOCS3 expression, can lead to excessive STAT activation, contributing to pathologies such as chronic inflammation or immune evasion in tumors (Croker et al., 2008). Furthermore, the JAK/STAT pathway interacts with other signaling pathways to fine-tune macrophage polarization. For instance, the PI3K/Akt/mTOR pathway can cross-talk with JAK/STAT signaling to modulate M1 and M2 polarization. Activation of PI3K/Akt enhances STAT1 phosphorylation and promotes M1 polarization. Meanwhile, mTOR signaling modulates STAT6 activity in a context-dependent manner, either enhancing or suppressing it to influence M2 polarization (Saleiro and Platanias, 2015). Additionally, the NF-κB pathway, which is crucial for inflammatory responses, can synergize with STAT1 to amplify the expression of pro-inflammatory genes in M1 macrophages (Yu et al., 2020). Conversely, STAT6 can interact with NF-κB to suppress its activity, promoting an anti-inflammatory environment conducive to M2 polarization (Shen and Stavnezer, 1998). These interactions highlight the complexity of macrophage polarization, where multiple signaling pathways converge to regulate the balance between pro-inflammatory and anti-inflammatory responses.

#### 2.2.3 PI3K/Akt signaling pathway

The PI3K/Akt signaling pathway is an important intracellular transduction pathway, activated by growth factors, cytokines, or stimulants such as LPS and IL-4 (Khezri et al., 2022). Phosphoinositol-3 kinase (PI3K) consists of a catalytic subunit (p110) and a regulatory subunit (p85). As a lipid kinase, its catalytic activity primarily involves phosphorylating phosphatidylinositol. The p85 subunit contains SH2 domains that bind phosphorylated tyrosine residues on activated receptors (e.g., IL-4R), while its Ras-binding domain (RBD) mediates PI3K membrane localization (Comb William et al., 2012). Under the influence of stimulants, PI3K catalyzes t e phosphorylation of phosphatidylinositol bisphosphate (PIP2), generating phosphatidylinositol (3,4,5)-trisphosphate (PIP3), which promotes the recruitment and activation of Akt (protein kinase B) (Xie et al., 2019). Akt activation requires dual phosphorylation. Initially, PIP3 binds to its pleckstrin homology (PH) domain, anchoring Akt to the membrane. Subsequently, PDK1 phosphorylates Thr308 in the activation loop, and mTORC2 phosphorylates Ser473 in the hydrophobic motif (Dan et al., 2016). Akt has three isoforms—Akt1, Akt2, and Akt3—all of which are serine/threonine protein kinases with distinct functions (Adon et al., 2025). Akt1 deficiency impairs NF-κB-driven M1 polarization by reducing IKKβ phosphorylation, whereas Akt2 knockout mice exhibit defective IL-4-induced STAT6 activation and M2 gene expression (Guo et al., 2022a). Studies have shown that Akt1 plays an important role in the M1 polarization process, while Akt2 is crucial for signal transduction in the M2 polarization process, although the actual impact depends on a variety of factors (Vergadi et al., 2017). Notably, Akt2 phosphorylates and inhibits GSK3β, thereby stabilizing β-catenin to enhance PPARγ transcriptional activity in M2 macrophages (Fang et al., 2007). In addition, IL-10 significantly affects this pathway. Research has found that IL-10 can activate PI3K and subsequently mTORC1 (mammalian target of rapamycin complex 1) via Akt. mTORC1 is a key signaling complex that regulates cell growth and metabolism. In certain contexts, it may also promote M1 polarization (Ben-Sahra and Manning, 2017).

#### 2.2.4 Notch signaling pathway

The Notch signaling pathway, a highly conserved mechanism critical for cell fate determination and differentiation. It was first identified in 1914 through Thomas Hunt Morgan’s studies of Notch mutants in *Drosophila melanogaster* (Borggrefe and Oswald, 2009). This pathway plays an essential role in regulating developmental processes and maintaining tissue homeostasis in multicellular organisms. Signal transduction initiates when Delta or Jagged family ligands bind to Notch receptors on the cell surface. This binding induces a conformational change in the receptor, triggering proteolytic cleavage by the γ-secretase complex (Miyamoto and Weinmaster, 2009). The cleavage releases the Notch intracellular domain (NICD), which translocates to the nucleus and binds the transcription factor RBP-J (CSL/CBF1) (Borggrefe et al., 2016). Together, NICD and RBP-J form a transcriptional activation complex that drives the expression of downstream target genes, including members of the Hes and Hey families, which are instrumental in regulating cell differentiation, proliferation, and tissue maintenance (Iso et al., 2003).

Recent studies have highlighted the pivotal role of Notch signaling in macrophage polarization. Pathway activation promotes M1 polarization, enhancing pro-inflammatory responses and microbial defense (Lin et al., 2018). Conversely, inhibition of Notch signaling shifts polarization toward the M2 phenotype, which prioritizes anti-inflammatory functions and tissue repair (Tan et al., 2024). For example, astragalus polysaccharide has been shown to activate the Notch signaling pathway and induce pro-inflammatory M1 polarization, whereas capsaicin suppresses the pathway, reducing M1 polarization and favoring M2-associated anti-inflammatory activity (Lin et al., 2018). Furthermore, microRNAs (miRNAs) such as miR-125a, miR-99b, and miR-148a-3p act as key modulators of Notch-mediated macrophage polarization by regulating NICD stability and function (Wang et al., 2019a). NICD activity is transient and regulated by post-translational modifications. For instance, ubiquitination marks NICD for proteasomal degradation (Damgaard, 2021). This mechanism prevents excessive signaling, maintaining cellular equilibrium and supporting tissue development. Such precise regulation underscores Notch signaling’s role in macrophage plasticity and its broader immunological and developmental functions.

#### 2.2.5 Other signaling pathway

In addition to the previously described pathways, several other signaling cascades critically influence macrophage polarization by modulating macrophage function and polarization states. Among these, the JNK, peroxisome proliferator-activated receptor gamma (PPARγ), interferon regulatory factor (IRF), TGF-β/SMAD, and phosphoinositide 3-kinase gamma (PI3Kγ) pathways have emerged as key regulators. The JNK signaling pathway, a member of the Mitogen-Activated Protein Kinase (MAPK) family, plays a crucial role in cellular processes such as proliferation, differentiation, and apoptosis (Zeke et al., 2016). In metabolic disorders like obesity, JNK activation is triggered by inflammatory mediators and lipid imbalances (Garg et al., 2021). Activation of JNK skews macrophage polarization toward the pro-inflammatory M1 phenotype, underscoring its role in immune regulation and metabolic homeostasis (Zhou et al., 2014). The PPARγ signaling pathway is another central regulator of macrophage polarization, primarily promoting the anti-inflammatory M2 phenotype. By modulating lipid metabolism and inflammation, PPARγ facilitates the expression of genes such as Arg1 and CD163, which enhance anti-inflammatory responses and tissue repair (Yao et al., 2018). Furthermore, PPARγ decreases pro-inflammatory activity in M1 macrophages by inhibiting the NF-κB pathway, emphasizing its importance in maintaining immune balance and resolving inflammation (Kim et al., 2017).

Similarly, members of the IRF family play crucial roles in macrophage polarization. IRF5 promotes M1 polarization by upregulating pro-inflammatory genes, while IRF4 fosters M2 polarization by activating transcriptional programs associated with anti-inflammatory responses and tissue remodeling (Krausgruber et al., 2011). The TGF-β/SMAD pathway is vital for coordinating immune responses and promoting tissue repair through macrophage polarization. When activated, TGF-β signaling induces the phosphorylation of SMAD2 and SMAD3 transcription factors, which then translocate to the nucleus to upregulate M2-associated genes such as Arg1 and Mgl2 (Garg et al., 2025). Conversely, SMAD7, an inhibitory SMAD, dampens this pathway to prevent excessive SMAD2/3 phosphorylation as part of a negative feedback loop (Zhao et al., 2000). Additionally, growth differentiation factor 11 (GDF11), upregulated in damaged tissues, binds to its receptor TGFβR1, enhancing SMAD2 phosphorylation and activating anti-inflammatory and pro-repair genes (Ma et al., 2021). Phosphorylated SMAD2 (P-SMAD2) interacts with other transcription factors in the nucleus, suppressing M1 markers like iNOS and CD86 while promoting anti-inflammatory effectors such as CD163, CXCR4, and Arg1, thus favoring M2 polarization (Bruce and Sapkota, 2012). The PI3Kγ pathway modulates macrophage polarization through Akt and mTOR. PI3Kγ inhibits NF-κB and activates C/EBPβ, driving immunosuppressive M2 polarization (Vergadi et al., 2017). Conversely, PI3Kγ inhibition enhances NF-κB activity and suppresses C/EBPβ, shifting macrophages toward a pro-inflammatory state. This reprogramming boosts CD8^+^ T-cell cytotoxicity and immune surveillance (Yuan et al., 2019).

### 2.3 The connection between macrophage polarization and diseases

These pathways and regulatory mechanisms collectively govern macrophage polarization through intricate signaling networks. Understanding these mechanisms is critical for defining macrophages’ roles in inflammatory, cancerous, metabolic, and neurological diseases (Jin et al., 2021). During early inflammation, macrophages predominantly polarize to the M1 phenotype. These cells release pro-inflammatory mediators that amplify inflammatory responses to external stimuli (Xia et al., 2023). While acute M1 activation is essential for host defense, persistent M1 activity drives pathological changes. Uncontrolled inflammatory factor release perpetuates chronic inflammation and exacerbates autoimmune diseases (Liu et al., 2014). To limit tissue damage, inflammation resolution involves macrophage apoptosis or M2 polarization. This shift suppresses excessive inflammation and promotes repair (Rodríguez-Morales and Franklin, 2023). M2 macrophages produce high levels of anti-inflammatory cytokines, fostering tissue repair, extracellular matrix remodeling, and angiogenesis while restoring homeostatic balance (Chen et al., 2023).

Macrophage plasticity also shapes cancer progression. TAMs initially adopt an M1 phenotype, which activates antitumor immunity and cytotoxicity. However, the evolving TME reprograms TAMs toward the M2 phenotype. M2 TAMs promote tumor proliferation, angiogenesis, and metastasis (Yang et al., 2020). This reprogramming is driven by multiple factors within the TME, including hypoxia, tumor-derived cytokines, and metabolic adaptations. Hypoxia stabilizes HIF-1α in TAMs, directly promoting M2 polarization through upregulation of Arg1 and VEGF, while suppressing pro-inflammatory M1 markers such as iNOS (Werno et al., 2010). Tumor-derived cytokines (IL-4, IL-10, TGF-β) activate STAT6 and SMAD pathways, reinforcing M2 immunosuppression (Mirlekar, 2022). Additionally, metabolic shifts in the TME, such as lactate accumulation and altered glucose metabolism, further skew macrophages toward the M2 phenotype via mTOR-dependent pathways or epigenetic modifications (Wiehagen et al., 2017). This phenotypic plasticity has spurred therapeutic strategies aimed at reprogramming TAMs toward the M1 phenotype or inhibiting M2 polarization. For instance, CSF-1R inhibitors and CD40 agonists have shown promise in preclinical and clinical trials by blocking M2-polarizing signals or reactivating M1-associated antitumor immunity (Li et al., 2022a). This plasticity has inspired therapies to re-polarize TAMs toward M1 or block M2 signals. In metabolic diseases, M1 macrophages drive insulin resistance, whereas M2 macrophages enhance insulin sensitivity and tissue repair (Guo et al., 2022b). Similarly, in neurological disorders, M1 activation exacerbates neuroinflammation, while M2 polarization supports neuroprotection and regeneration (Davis Michael et al., 2013).

As these examples illustrate, macrophage polarization dynamically adapts to disease-specific microenvironments, influencing pathogenesis and resolution. Additionally, macrophage polarization has been linked to other disorders, including atherosclerosis, tuberculosis, and chronic obstructive pulmonary disease (COPD) (Li et al., 2018). Understanding these mechanisms offers therapeutic insights, such as saponin compounds that modulate macrophage behavior. Importantly, the following section will classify saponins and explore their roles in macrophage polarization.

## 3 The therapeutic applications of saponins in regulating macrophage polarization

**FIGURE 3:**
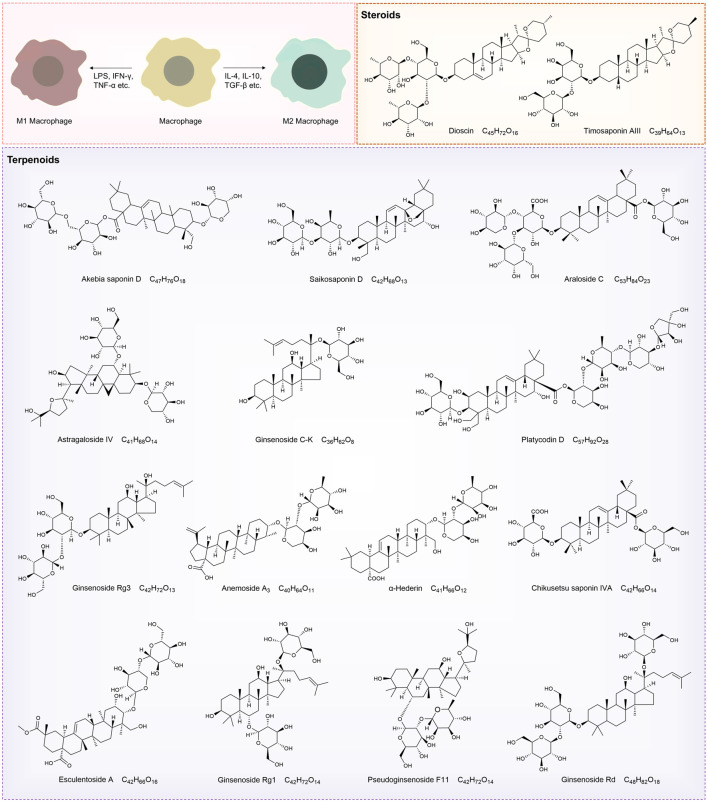
Structural diagrams of natural saponin compounds targeting macrophage polarization.

**FIGURE 4:**
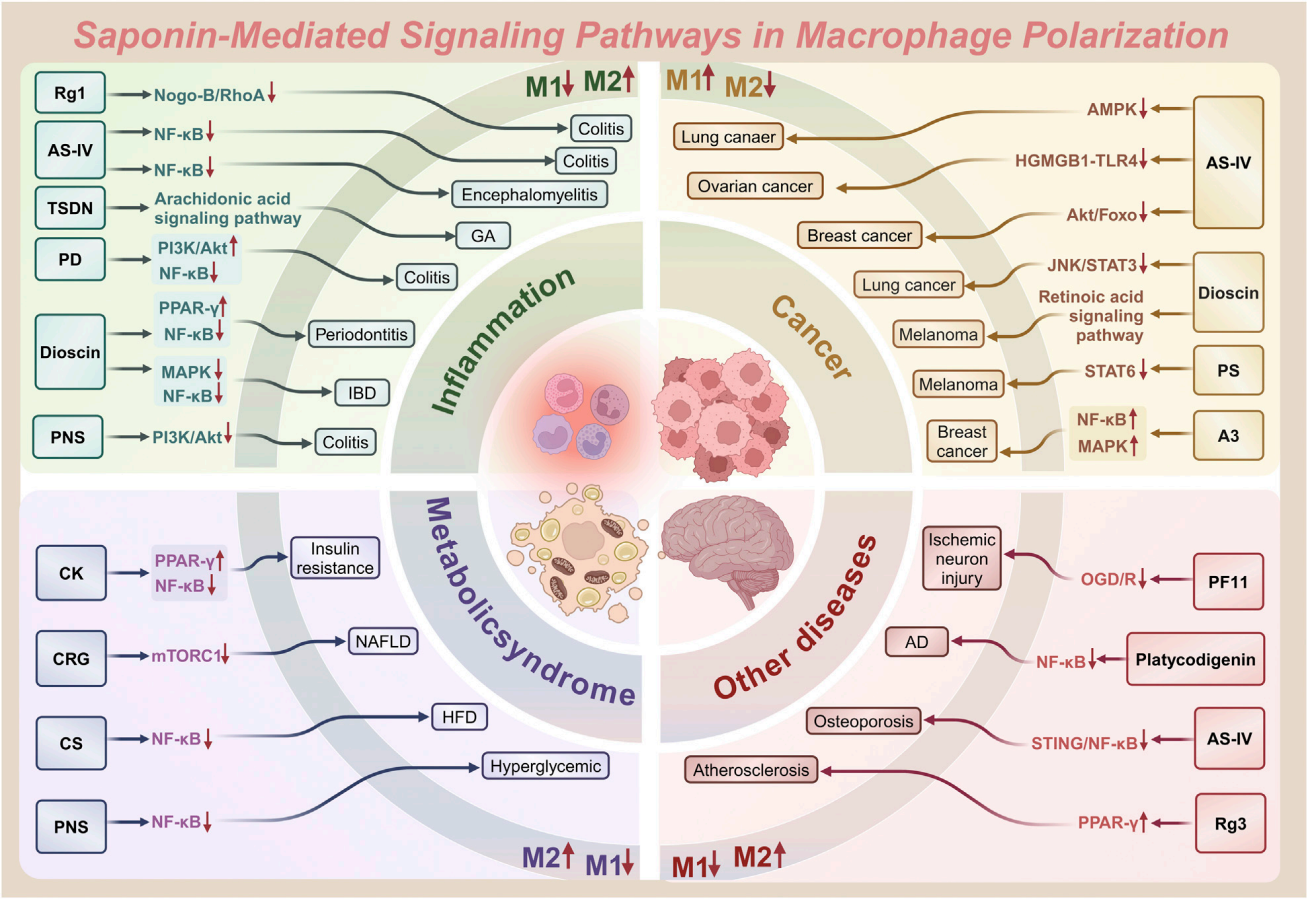
Saponin-mediated disease regulation: therapeutic intervention for diseases such as inflammation, cancer and more through multiple pathways targeting macrophage polarization (M1/M2).

Natural products—chemical entities derived from living organisms via primary or secondary metabolism—have been cornerstone therapeutic agents throughout human history (Mir et al., 2021). These compounds include alkaloids, terpenoids, flavonoids, and saponins. Their structural diversity and bioactivity make them indispensable in drug discovery and traditional medicine (Mir et al., 2023). Among these, saponins—a class of amphiphilic glycosides, consist of a triterpenoid or steroid aglycone (sapogenin) linked to sugar moieties. Their surfactant-like structure enables immunomodulatory and membrane-permeabilizing activities, driving significant research interest. This dual functionality allows saponins to interact with cholesterol-rich membranes and intracellular signaling pathways, establishing them as potent immune regulators (Mohi-Ud-Din et al., 2022). Macrophage polarization, as previously discussed, involves the intricate interplay of various signaling pathways. By binding to cell surface receptors, saponins modulate signaling pathways to regulate macrophage polarization (Brownlee, 2001; Xu and Núñez, 2023). Table 1 systematically summarizes key saponins, their mechanisms in macrophage polarization modulation, disease models, and therapeutic outcomes. Figure 3 illustrates saponin structural diversity, emphasizing chemical features essential for bioactivity. Figure 4 schematically outlines how saponins regulate macrophage phenotypes across diseases, bridging molecular mechanisms to clinical relevance. This section explores saponins’ therapeutic potential across diseases, analyzing their mechanisms and applications in halting disease progression (Table 1) (Figures 3, 4).

**TABLE 1:** Therapeutic significance and applications of saponin-mediated macrophage polarization in modulating immune responses.

Compound	Source	Disease	Methods	Dosage of administration	Mechanisms of treatment	Ref.
Ginsenoside Rg1	*Ginseng*	Colitis	Male specific pathogen-free (SPF) BALB/c mice	200 mg/kg	Reduced Rock1, RhoA, and Nogo-B levels; inhibited Nogo-B/RhoA signaling pathway activation	Long et al. (2022)
Ginsenoside Rg3	*Ginseng*	Atherosclerosis	Male ApoE^−/−^ mice	10 mg/kg	Promoted M2 macrophage polarization via a PPARγ-dependent mechanism; inhibited AGEs-induced M1 activation	Guo et al. (2018)
CK	*Ginseng*	Obesity-induced insulin resistance	C57BL/6N male	15, 30, 60 mg/kg	Reduced M1 inflammatory factors in obese mice macrophages; improved insulin resistance and glucose tolerance by upregulating PPARγ and inhibiting the TLR4/TRAF6/TAK1/NF-κB pathway	Wang et al. (2021a), Xu et al. (2022)
Ginsenoside Rd and Rg3	*Panax ginseng*	NAFLD	C57BL/6N male	100, 300 mg/kg	Exerted anti-inflammatory effects; promoted M2 polarization mediated by mTORC1	Choi et al. (2019)
CS	*Panax japonicas*	HFD	Male C57BL/6 mice	50, 100 mg/kg	Inhibited NF-κB signaling activation induced by an HFD; prevented adipose tissue macrophage accumulation and promoted M1 to M2 polarization by reducing phosphorylation levels and key ratios	Yuan et al. (2017)
PF11	*Panax pseudoginseng subsp*	Ischemic neuron injury	Primary cortical neurons from Sprague Dawley (SD) rat pups	10, 30, 100 μM	Attenuated the exacerbation of M1 macrophages and promoted the protective effect of M2 macrophages against OGD/R-induced primary neuronal injury	Hou et al. (2020)
AS-IV	*Astragali radix*	Lung cancer	Male C57BL/6 mice	40 mg/kg	Inhibited IL-4 and IL-13-induced M2 macrophage polarization; inhibited lung cancer progression and metastasis by partially blocking M2 polarization via the AMPK pathway	Xu et al. (2018)
AS-IV	*Astragali radix*	Breast cancer	Balb/c nude mice	40 mg/kg	Blocked TGF-β and the Akt/Foxo1 signaling pathway; inhibited M2 macrophage polarization	Yu et al. (2023b)
AS-IV	*Astragali radix*	Ovarian cancer	Human monocytic leukemia cells THP-1 and ovarian cancer cell line SKOV3	0.5–100 μg/mL	Suppressed expression of associated molecules (CD206, CCL24, PPARγ, Arg-1, IL-10) and the HMGB1-TLR4 signaling pathway; inhibited M2 macrophage polarization	Wang et al. (2021c)
AS-IV	*Astragali radix*	Colorectal Cancer	BALB/c female mouse	15 mg/kg	Reduced anti-inflammatory factors (TGF-β, IL-10, VEGF-A) and increased pro-inflammatory factors (IFN-γ, IL-12, TNF-α) in tumors; shifted macrophages from M2 to M1 phenotype, thereby inhibited tumor growth	Liu et al. (2020)
AS-IV	*Astragali radix*	Autoimmune encephalomyelitis	Female C57BL/6 mice	200 mg/kg	Inhibited neurotoxic M1 microglia/macrophages and blocked the TLR4/MyD88/NF-κB signaling pathway; protected neurons from apoptosis by promoting M2 phenotype and transforming astrocytes into the neuroprotective A2 phenotype	Yu et al. (2023a)
AS-IV	*Astragali radix*	IBD	Male SPF C57BL/6 J mice	50, 100 mg/kg	Suppressed STAT1 while enhancing STAT3 activation; inhibited M1 macrophage polarization and promoted M2 polarization	Tian et al. (2021)
AS-IV	*Astragali radix*	OP	Male C57BL/6J mice	10 mg/kg	Promoted M2 and inhibited M1 macrophage polarization in H_2_O_2_-activated macrophages; achieved by inhibiting the STING/NF-κB signaling pathway	Li et al. (2024)
Dioscin	*Dioscorea plants*	Periodontitis	Mouse macrophage cell line RAW264.7	4 μM	Inhibited pro-inflammatory M1 macrophages and promoted anti-inflammatory M2 macrophages by reducing NF-κB p65 and IκB phosphorylation, enhancing PPARγ expression, and activating the PPARγ/NF-κB pathway	Xiang et al. (2024)
Dioscin	*Dioscorea plants*	Ulcerative colitis	Male BALB/C mice	0.625, 1.25, 2.5 µM	Inhibited M1 and promoted M2 macrophage polarization in RAW264.7 cells; regulated glycolysis and fatty acid oxidation by suppressing mTORC1/HIF-1α and activating mTORC2/PPARγ pathways	Wu et al. (2021)
Dioscin	*Dioscorea plants*	IBD	Male C57BL/6 mice	40 mg/kg	Maintained intestinal barrier function in DSS-induced colitis; accomplished by increasing tight junction proteins (ZO-1, occludin, Muc-2) and inhibiting NF-κB, MAPK, and NLRP3 inflammasome pathways	Cai et al. (2021)
Dioscin	*Dioscorea plants*	Melanoma	Female C57BL/6 mice	60 mg/kg	Inhibited B16 cell migration and invasion; activated retinoic acid signaling to enhance Cx43 expression, reduced cancer stem cell markers and EMT, and shifted TAMs from M2 to M1 phenotype, enhancing phagocytic ability and pro-inflammatory cytokine secretion	Kou et al. (2017)
Dioscin	*Dioscorea plants*	Lung cancer	Male C57BL/6 mice	30, 60 mg/kg	Induced M2 to M1 macrophage polarization by inhibiting JNK and STAT3 pathways; suppressed TAM function, reduced tumor progression and metastasis, enhanced phagocytic ability, decreased IL-10, and increased IL-12/IL-10 ratio	Cui et al. (2020)
TSDN	*Dioscorea nipponica Makino*	GA	Male SPF-grade SD rats	160 mg/kg	Suppressed proliferation of M1-type macrophages; achieved by inhibiting COX-2, 5-LOX, mPGES-1, (LTB4), CYP4A, and PGE_2_ expression in the arachidonic acid signaling pathway	Zhou et al. (2023)
PD	*Platycodon grandifloras*	Colitis	Male C57BL/J mice	10 mg/kg	Reduced M1 markers and increased M2 markers in LPS-stimulated RAW 264.7 cells; activated the AMPK pathway, enhanced PI3K/Akt, and reduced NF-κB activation, resulting in decreased inflammation and improved intestinal integrity	Guo et al. (2021)
Platycodigenin	*Platycodon grandifloras*	Alzheimer’s Disease	ICR mice	0.01–50 µM	Downregulated pro-inflammatory molecules (IL-1β, TNF-α, IL-6, NO) and upregulated IL-10; reduced excessive phosphorylation of MAPK p38 and NF-κB p65, inhibited M1 microglia polarization, and promoted M2 phenotype	Yang et al. (2019b)
A3	*Pulsatilla chinensis*	Breast tumor	Female BALB/c mice	5, 10, 20 mg/kg	Induced M1 macrophage polarization from M0 type via the TLR4/NF-κB/MAPK pathway; enhanced TNF-α and IL-12 secretion, inhibited breast cancer cell proliferation and angiogenesis through the IL-12/VEGF axis, thereby suppressed tumor growth and angiogenesis	Yin et al. (2021)
PS	*Pulsatilla chinensis*	Melanoma	Male C57BL/6 mice	400 mg/kg	Inhibited STAT6 phosphorylation induced by IL-4; reduced M2 macrophage marker gene expression (Arg1, FIZZ1, Ym1, CD206), thereby inhibited M2 polarization	Yang et al. (2023)
PNS	*Panax notoginseng*	Colitis	SD rats	50, 100 mg/kg	Reduced inflammatory responses and oxidative stress by inhibiting the PI3K/AKT pathway; protected colonic tissue, decreased cell apoptosis, inhibited M1 macrophage polarization, and promoted M2 polarization	Xu et al. (2019)
PNS	*Panax notoginseng*	Hyperglycemic Condition	The human THP-1 cell	20, 40, 60 ug/mL	Downregulated the NF-κB pathway to promote M2 macrophage polarization; high-dose treatment significantly increased M2 proportion and Ym1, Arg 1 mRNA expression, enhanced Arg 1 protein, and reduced IκB-α phosphorylation and NF-κB p50/p65 expression in high-glucose conditions	Zhao et al. (2018)
TSAIII	*Anemarrhena asphodeloides*	Colitis	Male C57BL/6 mice	2, 5 mg/kg	Inhibited NF-κB activation and pro-inflammatory cytokines (IL-1β, TNF-α, IL-6) in TNBS-induced colitis; increased IL-10, inhibited Th17 cell differentiation, and promoted Treg cell differentiation in the colonic lamina propria	Lim et al. (2015)
ASD	*Dipsacus asper*	inflammation	RAW264.7 cells	5, 10, 20 µM	Reduced inflammatory mediator production (NO, PGE_2_) and expression of DNMT3b, iNOS, IL-6, TNF-α; inhibited the IL-6-STAT3-DNMT3b axis and activated the Nrf2 pathway	Luo et al. (2022)
EsA	*Phytolacca esculenta Van Houtte*	Lung Cancer	Human myeloid leukemia mononuclear cells (THP-1)	20 µM	Downregulated mRNA levels of M2 markers CD206 and PPARγ; inhibited IL-6 expression and STAT3 phosphorylation, thereby attenuated IL-13/IL-4-induced M2 macrophage polarization	Yanhong et al. (2024)
Saikosaponin D	*Bupleurum falcatum*	Pancreatic Cancer	Female C57BL/6 mice	1 mg/kg	Reduced phosphorylated STAT6 levels and inhibited the PI3K/AKT/mTOR pathway; decreased polarization of M2-type macrophages	Xu et al. (2023)
AsC	*Aralia elata (Miq.) Seem*	Atherosclerosis	Male ApoE^−/−^ mice	20 mg/kg	Increased Sirt1 expression; reduced plaque area and lipid accumulation in atherosclerotic mice, while promoted M2 macrophage polarization	Luo et al. (2020)
α-Hederin	*Hedera or Nigella sativa*	Sepsis	Male C57BL/6 mice	0.3, 3 mg/kg	Reduced TNF-α and IL-6 levels, decreased ALT and AST activity; inhibited M1 macrophage markers (CD86, iNOS), increased M2 markers (CD206), and regulated NF-κB pathway by reducing p-p65/p65 ratio and elevating IκB levels	(Zeng and Zhao, 2023)

### 3.1 Ginsenosides: multifaceted modulators of macrophage polarization

#### 3.1.1 Macrophage polarization in the context of metabolic disorders

Obesity and its related metabolic disorders, including type 2 diabetes and non-alcoholic fatty liver disease (NAFLD), are significant global health challenges (Younossi et al., 2018). Among ginsenoside CK (CK) has shown therapeutic potential for type 2 diabetes (Lumeng et al., 2007). A study by Xu et al. found that CK supplementation in a high-fat diet (HFD)-induced obese mouse model (C57BL/6J) alleviated insulin resistance by activating peroxisome proliferator-activated receptor γ (PPARγ). CK reduced M1-type inflammatory factors in a dose-dependent manner, improving insulin sensitivity and glucose tolerance while modulating the PPARγ/NF-κB axis to decrease systemic inflammation (Xu et al., 2022). Additionally, CK significantly lowered pro-inflammatory cytokines like MCP-1, TNF-α, and IL-1β in obese mice’s serum and adipose tissue, while enhancing anti-inflammatory factors in adipose tissue, highlighting its role in suppressing inflammation and promoting a favorable immune response (Sharma and Lee, 2020; Wang et al., 2021b).

Beyond CK, Korean red ginseng (RG) derivatives have shown efficacy against NAFLD—a key component of metabolic syndrome (Younossi et al., 2018). Korean red ginseng (RG), following solid-state fermentation with *Cordyceps sinensis* to produce fermented RG (CRG), has demonstrated enhanced bioactivity through increased concentrations of active ginsenosides Rd and Rg3. CRG significantly reduced the expression of M1 macrophage markers, including Ccl2, Ccl5, Il-1β, Il-6, iNos, and Tnf-α, by inhibiting the mTORC1 signaling pathway. Concurrently, CRG upregulated anti-inflammatory M2 macrophage markers, such as CD163 and IL-10, thereby promoting macrophage polarization toward the M2 phenotype. Through these mechanisms, CRG effectively alleviated the inflammatory response associated with NAFLD by modulating macrophage polarization and suppressing mTORC1 activation (Choi et al., 2019).

In complement to ginsenosides, Yuan et al. demonstrated that Chikusetsu saponin IVa (CS) reduced macrophage infiltration and polarization toward the M1 phenotype, which is characterized by the secretion of pro-inflammatory cytokines such as TNF-α, IL-6, and MCP-1 (Yuan et al., 2017). CS reduced M1 macrophage infiltration and pro-inflammatory cytokine secretion (TNF-α, IL-6, MCP-1), while increasing M2 macrophages. This polarization shift improved metabolic outcomes (Yuan et al., 2017; He et al., 2021a). CS also suppressed inflammasome components, including IL-1β, Caspase-1, NLRP3, and ASC. Additionally, CS attenuated NF-κB signaling by reducing the phosphorylation of NF-κB, IKK, and IκB, thereby limiting NF-κB nuclear translocation and the subsequent expression of pro-inflammatory genes. By promoting M2 polarization and suppressing inflammatory pathways, CS demonstrated potent anti-inflammatory effects in HFD models (Yuan et al., 2017).

#### 3.1.2 The neuroprotective role of macrophage polarization

Cerebral ischemia, a prevalent acute cerebrovascular condition, poses a significant health challenge for both the elderly and younger populations (Yang et al., 2022). Pseudoginsenoside-F11 (PF11), a dammarane-type ginsenoside from American ginseng (*Panax pseudoginseng subsp*)—has demonstrated neuroprotective effects in permanent and transient cerebral ischemia models (Hou et al., 2020). Studies show that ischemic neuronal conditioned medium, induced by oxygen-glucose deprivation/reoxygenation (OGD/R), drives macrophages toward the M1 phenotype. This polarization is characterized by elevated NOS2 expression and reduced Mrc1 expression (Liu et al., 2022). Treatment with PF11 (30 and 100 μM, with the latter showing stronger effects) significantly decreased NOS2 expression while increasing Mrc1 expression. Immunofluorescence analysis confirmed that PF11 reduced iNOS fluorescence intensity (an M1 marker) in macrophages and enhanced CD206 fluorescence intensity (an M2 marker). These findings indicate that PF11 promotes M2 macrophage polarization, enhancing neuroprotective effects on ischemic neurons and counteracting M1-mediated damage (Hou et al., 2020; Liu et al., 2022).

#### 3.1.3 Therapeutic implications of macrophage polarization in colitis

Macrophage polarization is pivotal in the pathogenesis of colitis, a chronic autoimmune inflammatory disease (Zhang et al., 2022). Long et al. investigated the therapeutic efficacy of ginsenoside Rg1 in a dextran sulfate sodium (DSS)-induced recurrent colitis mouse model, revealing its ability to inhibit macrophage activation. Rg1 significantly downregulates pro-inflammatory M1 markers—such as CD11b^+^ F4/80^+^ iNOS^+^ and TLR4^+^—thereby reducing pro-inflammatory cytokine production, including IL-6, IL-33, CCL-2, and TNF-α. Concurrently, Rg1 upregulates anti-inflammatory M2 markers—like CD11b^+^ F4/80^+^ Arg1^+^ and CD206^+^—enhancing their polarization and increasing IL-4 and IL-10 expression (Long et al., 2022). Furthermore, Rg1 modulates the Nogo-B/RhoA signaling pathway, suppressing RhoA-mediated inflammatory signaling to promote M2 macrophage polarization (Ajoolabady et al., 2024). Additionally, Rg1 improved gut microbiota composition, regulating macrophage-microbiota interactions for intestinal immune homeostasis (Zhou et al., 2016).

Macrophage polarization similarly drives atherosclerosis, a chronic inflammatory disease exacerbated by hyperglycemia in diabetic patients (Zhou et al., 2014). In diabetic atherosclerosis models, ginsenoside Rg3 alleviated disease progression by promoting M2 polarization (Guo et al., 2018). Under hyperglycemic conditions, advanced glycation end products (AGEs) induce macrophage M1 polarization, resulting in increased pro-inflammatory cytokines like IL-6 and TNF-α (Zhao et al., 2023). Ginsenoside 20(S)-Rg3, a PPARγ agonist, activated PPARγ signaling to suppress AGEs-induced M1 polarization while enhancing M2 polarization. This shift increased anti-inflammatory cytokines (IL-10, TGF-β) and reduced pro-inflammatory molecules, mitigating plaque inflammation and macrophage infiltration (Han et al., 2006). *In vivo* studies indicate that 20(S)-Rg3 enhances plaque stability and reduces plaque burden, with co-administration of the PPARγ antagonist GW9662 abolishing these effects, underscoring the importance of PPARγ activation for Rg3’s therapeutic benefits (Zhang and Chawla, 2004; Han et al., 2006).

In brief, ginsenosides exhibit significant therapeutic potential for various diseases, including neurodegenerative disorders, metabolic syndrome, and cancer. Their mechanisms of action primarily involve activating the PPARγ signaling pathway, suppressing NF-κB and NLRP3 inflammasome signaling, and modulating the mTORC1 pathway. These effects reduce inflammation, promote tissue repair, and restore immune homeostasis. Additionally, other saponins, such as those from Astragalus membranaceus IV, also play critical roles in regulating macrophage polarization. The therapeutic potential of these compounds will be explored in the following section.

### 3.2 Astragaloside IV: anti-cancer and anti-inflammatory agent with osteoprotective properties

#### 3.2.1 Mechanisms of macrophage polarization in cancer inhibition

Astragaloside IV (AS-IV), a naturally derived saponin isolated from the medicinal plants *Astragalus membranaceus*, has garnered attention for its potent anti-cancer properties (Yu et al., 2023a). Xu et al. demonstrated that AS-IV notably inhibits the polarization of M2 macrophages induced by IL-4 and IL-13, evidenced by decreased expression of CD206 and associated genes. This suppression mitigates the pro-tumor effects of M2 macrophages (Xu et al., 2018), including the invasion, migration, and angiogenesis of A549 and H1299 lung cancer cells when exposed to M2-conditioned medium (M2-CM). Mechanistically, AS-IV downregulates AMPKα activation in M2 macrophages, and silencing AMPKα partially rescinds this effect. Complementary *in vivo* studies further reveal that AS-IV significantly curtails lung cancer growth and diminishes metastatic spread (Cheng et al., 2014; Xu et al., 2018).

In colorectal cancer (CRC), AS-IV has shown promise by re-educating TAMs. Liu et al. observed that AS-IV dramatically reduced the expression of M2 macrophage markers, such as CD206, in bone marrow-derived macrophages (BMDMs) initially polarized to the M2 phenotype, while upregulating M1 macrophage markers, including MHC II (Liu et al., 2020). Gene and protein expression analyses corroborated these findings, as AS-IV downregulated M2-associated genes (Arg1, Mrc1) and upregulated M1-associated genes (IL-12, Nos2). Correspondingly, *in vivo* experiments using a CRC mouse model showed that AS-IV not only inhibited tumor growth but also remodeled the macrophage population within the TME by reducing the proportion of M2 macrophages and increasing M1 macrophages. Additionally, AS-IV downregulated anti-inflammatory cytokines such as TGF-β, IL-10, and VEGF-A while upregulating pro-inflammatory cytokines including IFN-γ, IL-12, and TNF-α, thereby fostering a more immunologically active tumor milieu (Liu et al., 2020).

Further, studies by Wang et al. highlighted AS-IV’s regulatory impact on M2 macrophages in ovarian cancer. The researchers uncovered that AS-IV inhibits M2 polarization by targeting the high mobility group box 1 (HMGB1) and TLR4 signaling pathways (He et al., 2013; Zhou et al., 2024). During co-culture with ovarian cancer cells, HMGB1 and TLR4 expression was significantly elevated in M2 macrophages, while AS-IV treatment reversed this effect. Through suppression of the HMGB1/TLR4 axis, AS-IV reduced M2 macrophage-mediated production of pro-tumor factors such as TGF-β, matrix metalloproteinase 9 (MMP-9), and IL-10. This inhibition, in turn, suppressed the proliferation, migration, and invasion of ovarian cancer cells. Notably, supplementation with exogenous HMGB1 partially abrogated AS-IV’s inhibitory effects, substantiating the central role of HMGB1 signaling in AS-IV’s mechanism of action (Wang et al., 2021c).

AS-IV has also demonstrated efficacy in breast cancer by modulating macrophage polarization. Yu et al. employed qRT-PCR, Western blot, and flow cytometry to show that treatment with AS-IV suppressed M2 macrophage polarization in IL-4- and IL-13-stimulated M0 macrophages, as evidenced by reduced expression of CD206 and CD163 (Yu et al., 2023b). In addition, AS-IV curtailed the secretion of cytokines associated with M2 macrophage activity, such as IL-10 and MMP-9. Mechanistic studies revealed that AS-IV inhibits TGF-β-mediated phosphorylation of Akt and Foxo1, which is critical for driving M2 polarization. Adding exogenous TGF-β or the Akt activator SC79 reversed AS-IV’s inhibitory effects, leading to heightened breast cancer cell proliferation, migration, and invasiveness. These findings underscore the importance of the TGF-β/Akt/Foxo1 axis in regulating M2 polarization and its modulation by AS-IV (Yu et al., 2023b).

#### 3.2.2 Role of macrophage polarization activators in colitis and meningomyelitis

AS-IV has shown promise as a therapeutic agent for inflammatory bowel disease (IBD) through its immunomodulatory effects (Vieujean et al., 2025). In a DSS-induced colitis model, AS-IV alleviated symptoms such as weight loss and increased disease activity index (DAI), while reducing colon length (Tian et al., 2021). It facilitated a macrophage phenotypic shift from pro-inflammatory M1 to anti-inflammatory M2, decreasing cytokines like IL-1β and TNF-α while increasing IL-10 and TGF-β in colonic tissues. This shift was mediated by downregulating M1-related genes under LPS and IFN-γ stimulation and enhancing M2-related gene expression in BMDMs, involving inhibition of STAT1 phosphorylation and activation of STAT3 signaling, confirmed by molecular docking experiments that showed AS-IV binding to STAT1 (Tian et al., 2021; Li et al., 2023).

Beyond IBD, AS-IV exhibits neuroprotective effects in experimental autoimmune encephalomyelitis (EAE) models by modulating macrophage polarization. It suppressed M1 activation (CD16/32^+^, CD11c^+^, IL-12^+^) and promoted M2 markers (CD206^+^, IL-10^+^). These effects were achieved by inhibiting the TLR4/MyD88/NF-κB pathway, which reduced pro-inflammatory cytokines (IL-1β, TNF-α) and elevated IL-10 (Cheng et al., 2014). Notably, exogenous HMGB1 supplementation partially restored pro-inflammatory signaling, underscoring the interplay between HMGB1 and macrophage polarization in neuroinflammation (Chen et al., 2024).

#### 3.2.3 Macrophage polarization as a protective mechanism in osteoporosis

Osteoporosis (OP) is a metabolic bone disease characterized by progressive bone loss and fragility, with macrophage senescence identified as a key factor in its pathology (Khandelwal and Lane, 2023). AS-IV, a natural saponin derived from Astragalus species, has shown significant therapeutic potential by targeting macrophage senescence and polarization. Li et al. demonstrated that AS-IV inhibits macrophage senescence through downregulation of markers such as p21, p53, and p16, thereby delaying cellular aging. Additionally, AS-IV suppresses the STING/NF-κB signaling pathway to promote a shift from pro-inflammatory M1 to anti-inflammatory M2 macrophages (Li et al., 2021). In a hydrogen peroxide (H_2_O_2_)-induced senescence model, AS-IV decreased M1-related pro-inflammatory genes (TNF-α, iNOS) and increased M2-related anti-inflammatory markers (CD163, CD206). This polarization shift alleviates inflammation and enhances osteogenic differentiation of bone marrow mesenchymal stem cells (BMSCs), promoting bone repair. AS-IV also improves mitochondrial function in macrophages by reducing ROS and increasing mitochondrial membrane potential, further supporting M2 polarization and bone health (Li et al., 2024).

In summary, this section highlights the therapeutic potential of AS-IV in managing OP by targeting macrophage senescence and polarization. By regulating key signaling pathways like STING/NF-κB, AS-IV alleviates inflammation and enhances bone regeneration. Alongside its roles in cancer therapy and inflammatory modulation, AS-IV exemplifies the versatility of Astragalus-derived saponins in addressing various pathological conditions. The following section will explore the pharmacological properties of dioscin saponins and their role in modulating macrophage polarization to combat inflammation and tumors.

### 3.3 Dioscin: impact on macrophage polarization to combat inflammation and tumors

#### 3.3.1 Modulation of macrophage polarization in inflammation and arthritis

Dioscin, a steroidal saponin derived from plants such as Dioscorea species, has demonstrated remarkable anti-inflammatory and immunomodulatory properties (Wu et al., 2021). Xiang et al. investigated dioscin’s role in treating inflammatory diseases, particularly periodontitis. Their study focused on dioscin’s effects on macrophage polarization induced by LPS from *Porphyromonas gingivalis* (P.g.) (Xiang et al., 2024). Using the RAW264.7 macrophage cell line as an *in vitro* model, it is important to note that while RAW264.7 cells are widely used for their convenience and homogeneity, they exhibit distinct genetic and functional differences from primary human macrophages. For instance, these cells lack the full spectrum of human macrophage polarization markers (CD14/CD16 subsets) and may show altered cytokine secretion under inflammatory stimuli, limiting direct clinical extrapolation (Chen et al., 2015). Their findings revealed that dioscin activates peroxisome proliferator-activated receptor γ (PPARγ) while inhibiting the nuclear factor κB (NF-κB) signaling pathway. Specifically, dioscin reduces NF-κB p65 phosphorylation, prevents IκB degradation, and upregulates PPARγ expression (Yan and Wan, 2021). As a result, dioscin suppresses pro-inflammatory M1 macrophage polarization and enhances anti-inflammatory M2 macrophage polarization, underscoring its potential as a therapeutic agent for managing periodontitis.

Building on the investigations into dioscin’s effects on macrophage polarization in periodontitis, further research has explored its therapeutic potential in other inflammatory conditions. Cai et al. further explored the therapeutic potential of dioscin in IBD (Cai et al., 2021). Their findings revealed that dioscin reduces the levels of pro-inflammatory cytokines, such as tumor necrosis factor α (TNF-α) and interleukin 1β (IL-1β), while increasing the secretion of the anti-inflammatory cytokine IL-10 (Cai et al., 2021). Immunohistochemistry and immunofluorescence analyses showed dioscin decreases M1 marker CD80 and increases M2 marker CD206 in colonic tissues, thereby alleviating inflammation (Yan and Wan, 2021). Similarly, Wu et al. observed that dioscin inhibits M1 macrophage polarization by suppressing the mTORC1/hypoxia-inducible factor-1α (HIF-1α) signaling pathway. This inhibition is characterized by reduced glucose uptake, lactate secretion, and expression of key glycolytic enzymes, including HK-2, PKM2, and LDHA. Concurrently, dioscin activates the mTORC2/PPARγ signaling pathway, promoting fatty acid oxidation (FAO) to provide the energy required for M2 macrophage polarization. These findings establish a mechanistic basis for the use of dioscin in treating ulcerative colitis (Furfaro et al., 2021; Han et al., 2021).

In addition to its role in treating inflammatory conditions such as periodontitis and IBD, dioscin is also being explored for its potential in managing diseases like gouty arthritis (GA). Dioscorea nipponica Makino (TSDN), which contains dioscin, has shown promise in GA, a condition driven by the oversaturation of uric acid in the blood and the deposition of monosodium urate (MSU) crystals in the joints (Furfaro et al., 2021). Zhou et al. examined the effects of the total saponin fraction from TSDN on macrophage polarization in a GA model using RAW264.7 cells stimulated with LPS and MSU. TSDN treatment significantly inhibited M1 macrophage proliferation, as evidenced by reduced expression of pro-inflammatory markers such as cyclooxygenase-2 (COX-2), 5-lipoxygenase (5-LOX), and leukotriene B4 (LTB4). Furthermore, TSDN increased the expression of the anti-inflammatory enzyme microsomal prostaglandin E synthase-1 (mPGES-1) and enhanced M2 macrophage polarization by modulating the arachidonic acid signaling pathway. These effects reduce apoptosis and inflammation, supporting TSDN’s use in slowing GA progression (Zhou et al., 2023).

#### 3.3.2 Targeting macrophage polarization in melanoma therapy and lung cancer

Beyond its well-established anti-inflammatory effects, dioscin, a steroidal saponin derived from yam, has gained significant attention in recent years due to its potent anti-tumor properties. In the TME, TAMs predominantly adopt the M2 phenotype, which supports tumor progression, immune evasion, and metastasis (Mantovani and Locati, 2013). Conversely, M1-type TAMs exhibit anti-tumor activity by enhancing immune responses and inhibiting tumor growth. Thus, reprogramming TAMs from M2 to M1 or boosting M1 activity is crucial for cancer therapy (Kou et al., 2017). Li et al. demonstrated that dioscin suppresses tumor progression by upregulating connexin 43 (Cx43), a key regulator in the TME. Mechanistically, dioscin enhances both the expression and transport function of Cx43 via the retinoic acid pathway. This promotes a macrophage polarization shift from M2 to M1, marked by increased M1 markers (iNOS, CCR7) and decreased M2 markers (CD206, Arg-1). This transition reverses the epithelial-mesenchymal transition (EMT) in melanoma cells, a process driving tumor invasion. Additionally, cytokines from dioscin-reprogrammed macrophages—including IL-1β, TNF-α, and IL-6—suppress B16 melanoma cell migration, reduce cancer stem cell markers, and inhibit EMT potential (Shi et al., 2022).

In a subcutaneous lung cancer model, Cui et al. demonstrated that dioscin induces a macrophage polarization shift from the M2 phenotype to the M1 phenotype in both BMDMs and RAW264.7 cells. This process was mediated through the suppression of the STAT3 and JNK signaling pathways (Cui et al., 2020). Treatment with dioscin resulted in increased expression of M1-associated markers, such as NOS2 and IL-6, alongside a notable reduction in M2 markers, including Arg-1 and CD206. Additionally, dioscin inhibited the secretion of the immunosuppressive cytokine IL-10 by M2 macrophages, thereby enhancing macrophage phagocytic activity. *In vivo* experiments further confirmed that dioscin reduced the proportion of M2 macrophages in the TME, leading to significant suppression of tumor growth and metastasis. By promoting macrophage polarization towards the M1 phenotype, dioscin not only amplifies anti-tumor immune responses but also restricts tumor cell migration, metastasis, and angiogenesis (Cui et al., 2020).

In brief, dioscin exhibits profound anti-tumor and anti-inflammatory effects by modulating macrophage polarization, suppressing tumor-supportive pathways, and enhancing immune responses. Through its regulation of key signaling pathways such as JAK/STAT, mTORC1/HIF-1α, and mTORC2/PPARγ, dioscin effectively inhibits tumor progression while alleviating inflammation and promoting tissue repair. Similar to dioscin, other saponins, such as platycodin, also exhibit promising therapeutic potential. The application of platycodin in specific disease models will be explored in the subsequent section.

### 3.4 Platycodin D: facilitating disease prevention and treatment via macrophage polarization

Platycodin D (PD), a bioactive saponin extracted from Platycodon grandiflorum, has demonstrated significant anti-inflammatory properties (Li et al., 2022c). Recent studies by Guo et al. revealed that PD modulates macrophage polarization, suppressing the pro-inflammatory M1 phenotype while promoting the anti-inflammatory M2 phenotype in a DSS-induced mouse model of colitis (Guo et al., 2021). Specifically, *in vivo* experiments showed that PD treatment decreased the expression of M1 markers, such as iNOS and CD86, while upregulating M2 markers, including Arg1 and CD206. Flow cytometry revealed reduced M1 and increased M2 macrophage proportions after PD treatment. *In vitro* studies with LPS-stimulated RAW264.7 cells corroborated these findings: PD decreased M1 markers and elevated M2 markers. Mechanistically, PD activates AMPK, which promotes PI3K/Akt signaling and inhibits NF-κB, thereby driving M2 polarization and anti-inflammatory effects (He et al., 2023).

Beyond intestinal inflammation, platycodigenin, a derivative of Platycodon grandiflorum, has shown promise in the treatment of Alzheimer’s disease (AD). Yang et al. found that platycodigenin exerts anti-inflammatory effects by modulating the p38 MAPK and NF-κB signaling pathways. In LPS-stimulated BV2 microglial cells, platycodigenin reduced the expression of pro-inflammatory factors, such as TNF-α and IL-1β, while increasing levels of the anti-inflammatory cytokine IL-10. This shift was accompanied by a suppression of M1 microglial polarization, marked by decreased Cox2 expression, and an enhancement of M2 polarization, indicated by increased Ym1/2 and CD206 expression (Yang et al., 2019b). Furthermore, platycodigenin restored the expression of PPARγ, which is critical for M2 polarization but is typically suppressed by LPS stimulation. In neurons treated with amyloid-beta (Aβ), platycodigenin promoted neurite outgrowth and improved neuronal survival, highlighting its potential to mitigate neurodegeneration associated with AD (Yang et al., 2019b).

Collectively, these findings underscore the ability of PD and its derivatives to regulate macrophage polarization through pathways such as PI3K/Akt, p38 MAPK, and NF-κB, thereby alleviating inflammation and contributing to the treatment of diseases like colitis and Alzheimer’s. This evidence establishes a foundation for further exploration of saponins, including PS, in disease prevention and treatment, as detailed in the next section.

### 3.5 Pulsatilla saponins: the contribution of macrophage polarization in disease prevention and treatment support

The therapeutic potential of Pulsatilla saponins (PS) in modern medicine has garnered significant attention, particularly in the context of anti-tumor treatments (Zhong et al., 2022). Research by Yang et al. demonstrated that PSs regulate macrophage polarization by inhibiting M2 polarization through the IL-4/STAT6 signaling pathway. Specifically, PSs suppress STAT6 phosphorylation and nuclear translocation, which downregulates the expression of M2 marker genes and effectively hinders melanoma cell proliferation and migration. *In vivo* experiments further revealed that PSs can significantly reduce lung tumor metastasis and lower the expression of M2 marker genes in tumor tissues. The critical role of STAT6 inhibition in the mechanism of action was validated through co-administration with a STAT6-specific inhibitor, highlighting the pathway’s centrality (Yang et al., 2023). In breast cancer studies, Yin et al. identified the saponin Anemoside A3 (A3) as a key modulator of macrophage function. A3 binds to Toll-like receptor 4 (TLR4), activating downstream MyD88 and TRAF6 signaling cascades, which in turn trigger the NF-κB and MAPK pathways. This activation leads to an increased expression of pro-inflammatory cytokines such as TNF-α and IL-12 in M1 macrophages. Notably, the IL-12 upregulation not only enhances the anti-tumor activity of macrophages but also inhibits tumor cell proliferation and angiogenesis through the suppression of VEGF. *In vivo*, A3 treatment was shown to significantly increase the proportion of M1 macrophages in tumor tissues, elevate IL-12 levels, and reduce VEGF expression, ultimately resulting in suppressed tumor growth and decreased angiogenesis (Yin et al., 2021).

In addition to their ability to regulate macrophage polarization via the STAT6 signaling pathway, PS also modulate immune responses through the activation of the TLR4/NF-κB/MAPK signaling cascades and the balanced expression of pro-inflammatory and anti-inflammatory cytokines. Similarly, PNS have emerged as potent modulators of macrophage polarization, exhibiting significant therapeutic potential in various inflammatory and disease contexts. The following section delves into the detailed mechanisms of action and broader applications of PNS in clinical and experimental settings.

### 3.6 Panax notoginseng saponin: targeting macrophage polarization for health maintenance

Panax notoginseng saponin (PNS), an active compound derived from the dried roots and rhizomes of Panax notoginseng in the Araliaceae family, has shown therapeutic potential across a range of conditions (Lu et al., 2020). In a DSS-induced experimental colitis model in rats, PNS demonstrated the ability to suppress the PI3K/Akt signaling pathway, reduce oxidative stress, and lower pro-inflammatory cytokines such as IL-6, IL-1β, and TNF-α. Concurrently, PNS upregulated the expression of the anti-inflammatory cytokine IL-10, promoting M2 macrophage polarization while reducing the M1 macrophage population. This dual modulation effectively alleviates colonic inflammation and tissue damage (Xu et al., 2019). Under hyperglycemic conditions, PNS further demonstrated anti-inflammatory effects by inhibiting the NF-κB signaling pathway. Specifically, PNS decreased IκBα phosphorylation, thereby suppressing NF-κB activation and the transcription of inflammatory genes. This inhibition promoted a macrophage shift toward the M2 phenotype, as evidenced by increased expression of M2 markers such as Ym1 and Arg1. The anti-inflammatory characteristics of M2 macrophages highlight PNS’s efficacy in mitigating inflammatory damage and modulating immune responses (Zhao et al., 2018).

Therefore, PNS holds significant clinical value in the management of inflammatory diseases such as colitis and cardiovascular conditions like atherosclerosis. Through its dual inhibition of the PI3K/Akt and NF-κB signaling pathways, PNS effectively arrests M1-mediated inflammation while promoting M2 polarization, reducing inflammatory damage, and providing protection against tumor progression.

### 3.7 Other saponins: applications across diverse domains

Beyond the previously discussed saponin compounds, emerging research has highlighted the therapeutic potential of other saponins in modulating macrophage polarization. For instance, Luo et al. demonstrated that Akebia saponin D (ASD) suppresses the IL-6/STAT3/DNMT3b axis while activating the Nrf2 pathway, leading to a reduction in inflammatory mediator production and inhibition of M1 macrophage polarization (Luo et al., 2022). Similarly, Lim et al. reported that Timosaponin AIII (TSAIII), a key component derived from the rhizome of *Anemarrhena asphodeloides* (AA), prevents LPS from binding to TLR4 on macrophage surfaces. This inhibition reduces the phosphorylation of IRAK1 and TAK1, as well as the degradation of IκBα, thereby suppressing NF-κB and MAPK signaling pathways and mitigating inflammatory responses (Lim et al., 2015).

In addition to their anti-inflammatory properties, certain saponins have shown promise in TME modulation. For example, Esculentoside A (EsA) has been found to inhibit M2 macrophage polarization by downregulating M2 markers such as CD206 and PPAR-γ, suppressing IL-6 expression, and blocking STAT3 phosphorylation. These actions consequently impair the migration and invasion of lung cancer cells (Yanhong et al., 2024). Saikosaponin D, on the other hand, decreases M2 macrophage polarization by inhibiting phosphorylated STAT6 and the PI3K/Akt/mTOR signaling pathway, while simultaneously increasing M1 macrophage proportions, thereby influencing the progression of pancreatic cancer. The ability of these saponins to modulate macrophage polarization and reduce inflammatory damage is further evidenced by their impact on markers such as Ym1 and Arg1, which are significantly elevated following treatment (Xu et al., 2023).

Beyond their applications in inflammation and oncology, saponins have demonstrated potential in cardiovascular protection and sepsis treatment. Araloside C (AsC), a cardioprotective triterpenoid, has been shown to reduce arterial plaque burden and lipid accumulation in macrophages induced by oxidized low-density lipoprotein (ox-LDL) in atherosclerotic mice. By activating Sirt1-mediated autophagy, AsC promotes M2 macrophage polarization, increases autophagosome formation, and regulates autophagy-associated protein expression (Luo et al., 2020). Likewise, α-Hederin, a monosaccharide chain saponin, has exhibited efficacy in sepsis models by reducing M1 macrophage markers such as CD86 and iNOS, while increasing the M2 marker CD206. This shift is accompanied by inhibition of the NF-κB signaling pathway, which alleviates lung and liver tissue damage, reduces serum alanine aminotransferase (ALT) and aspartate aminotransferase (AST) levels, decreases malondialdehyde (MDA) production, and enhances antioxidant markers such as superoxide dismutase (SOD) and glutathione (GSH) (Long et al., 2022).

In summary, the diverse biological activities of saponins underscore their broad therapeutic potential across inflammation, oncology, cardiovascular health, and sepsis. Notably, distinct saponins exhibit context-dependent efficacy due to variations in their molecular targets and signaling pathways (Passos et al., 2022a). For instance, CK primarily modulates the PPARγ/NF-κB axis to suppress M1 polarization in metabolic disorders, achieving a 40% reduction in adipose tissue inflammation (Xu et al., 2018), while AS-IV demonstrates superior anti-tumor effects by targeting AMPKα and HMGB1/TLR4 pathways, reducing M2 macrophage-mediated tumor growth by 50% in CRC models (Wu et al., 2015). In contrast, dioscin exerts dual anti-inflammatory and anti-tumor activity through mTORC1/HIF-1α inhibition and PPARγ activation, showing broader applicability in both colitis and melanoma. These findings highlight the central role of macrophage polarization in the mechanisms of saponin action. Furthermore, structural differences critically influence functional specificity: PD’s activation of AMPK/PI3K/Akt pathways makes it more effective in neuroinflammatory diseases like Alzheimer’s compared to CS, which primarily targets NLRP3 inflammasomes in metabolic inflammation. Moving forward, further research into the pharmacokinetics and safety profiles of these compounds will be critical to advancing their clinical applications.

## 4 Pharmacokinetics and safety of saponins

### 4.1 Pharmacokinetics of saponins

Pharmacokinetic analysis is a cornerstone of drug development, providing critical insights into the absorption, distribution, metabolism, and excretion (ADME) of therapeutic agents. By understanding these processes, researchers can optimize drug selection, refine dosage regimens, and enhance therapeutic efficacy while minimizing adverse effects. Moreover, pharmacokinetic studies enable the prediction of drug-drug interactions and potential synergistic or antagonistic effects. This section explores the pharmacokinetics of key saponin compounds discussed in Section 3 (Table 2), including ginsenosides, AS-IV, dioscin, PD, PS, and PNS.

**TABLE 2:** Pharmacokinetics of saponin compounds: absorption, distribution, metabolism, and excretion data across various animal models.

Saponin	Subject	Weight	Method	Dose (mg/kg)	C_max_ (mg/L)	AUC_0-∞_(mg·L^-1^·h)	T_max_(h)	T_half_(h)	Ref.
Ginsenoside Rg1 liposomes	SPF healthy adult SD rats	300 ± 20 g	Intratracheally instilled	1	29.45 ± 25.73	—	—	49.32	Liang et al. (2024)
Ginsenoside Rg1 solution	SPF healthy adult SD rats	300 ± 20 g	Intratracheally instilled	1	164.51 ± 30.77	—	—	42.8	Liang et al. (2024)
Ginsenoside Rg3	Male Wistar rats	200–250 g	Oral administration (PO)	50	81.6 ± 24.6	326 ± 36.1	2.33 ± 0.58	4.27 ± 1.35	Fan et al. (2016)
Ginsenoside Rg3	Walker 256 Tumor-bearing Rats	200–250 g	PO	50	36.4 ± 11.3	137 ± 51.7	1.83 ± 0.632	2.47 ± 0.975	Fan et al. (2016)
Ginsenoside Rd	Healthy adult Kunming mice or Wistar rats	18–20 g or 180–200 g	Intravenous injection (IV)	50	—	293.2 ± 279.4	—	12.83 ± 2.92	Sun et al. (2012)
CK	Healthy Chinese Volunteers	BMI: 19–24 kg/m^2^	Single-dose oral	25–800	175.1–1183.2	1225.9–9093.6	2.5–3.3	13.5–16.2	Chen et al. (2017)
CK	Healthy Chinese Volunteers	BMI: 19–24 kg/m^2^	Multiple-dose oral	100–400	1116.3–5055.8	8876.8–43889.3	2.5–3.8	23.4–37.3	Chen et al. (2017)
CS	Male Wistar rats	220 ± 20 g	PO	20	448.19 ± 87.48	1422.49 ± 682.97	0.33 ± 0.13	1.59 ± 0.25	Wang et al. (2016)
CS	Male Wistar rats	220 ± 20 g	Subcutaneous injections (SQ)	1	—	824.46 ± 290.63	—	5.42 ± 1.91	Wang et al. (2016)
AS-IV	Male Kunming mice	35–40 g	Oral gavage (IG)	30	59.2	289.8	4	1.85	He et al. (2018)
Dioscin	Healthy adult male SD rats	—	PO	121.8	38.24 ± 0.63	28,379.48 ± 2066.52	10.29 ± 1.19	12.79 ± 1.66	Zhang et al. (2015)
PS	Male SD rats	250 ± 20 g	PO	20	44.45 ± 22.40	73.00 ± 24.17	0.44 ± 0.17	1.32 ± 0.64	Shan et al. (2015)
A3	Male SD rats	220 ± 20 g	PO	—	17.8 ± 6.9	345.3 ± 42.7	24	13.8	Tian et al. (2018)
PNS (mainly Rb1)	Male SD rats	200 ± 20 g	IG	40	230.00 ± 132.21	5400.9 ± 1778.67	1.93 ± 0.98	16.16 ± 4.03	Fu et al. (2023)
PNS (mainly Rb1)	Male SD rats	200 ± 20 g	IV	10	10,420.61 ± 1800.68	195,186.6 ± 23,229.48	0.083 ± 0.00	16.01 ± 1.65	Fu et al. (2023)

Ginsenosides, as prominent bioactive components, have been extensively studied for their pharmacological and pharmacokinetic properties (Sun et al., 2012). For instance, ginsenoside Rg1, predominantly derived from Panax notoginseng, exhibits a range of therapeutic effects but suffers from poor oral bioavailability (1%–20%) (Liang et al., 2024). This limitation is attributed to its rapid degradation by intestinal microbiota and swift systemic clearance (Xu et al., 2003). To address this, Liang and colleagues developed a liposomal formulation of Rg1, leveraging liposomes as drug carriers to enhance stability, targeted release, and systemic recovery. Their experiments demonstrated that Rg1 exhibits robust linearity within the concentration range of 1–20 μg/mL, with stability maintained for up to 8 h and a high recovery rate (Mohanan et al., 2018). Similarly, ginsenoside Rd follows a two-compartment pharmacokinetic model after intravenous (IV) administration. It shows rapid distribution across tissues (notably in the lungs) and primary excretion via urine (Sun et al., 2012). Another ginsenoside, Rg3, has garnered attention for its potent antitumor activity. ​Rg3 is metabolized into Rh2, a pharmacologically active prodrug. However, He et al. reported that Rg3 exhibits low oral bioavailability, with absorption and metabolism differing significantly between tumor-bearing and healthy rats (Won et al., 2019; Fan et al., 2016). In contrast, ginsenoside K, a metabolite of Rg3, demonstrates favorable linear pharmacokinetics and has been confirmed to be safe in healthy volunteers (Sharma and Lee, 2020; Chen et al., 2017). This observed variability may stem from differences in intestinal microbiota composition, formulation strategies, and interspecies metabolic disparities between preclinical models and humans.

The pharmacokinetics of CHS-IVa, another saponin of interest, reveal distinct characteristics. Wang et al. observed that after oral administration (PO), CHS-IVa reaches peak plasma concentration within 0.35 ± 0.14 h, followed by a rapid decline below detectable levels within 24 h. After IV administration, its half-life was 1.59 ± 0.25 h, with an absolute bioavailability of only 8.63%—indicating poor gastrointestinal absorption. This low bioavailability is attributed to its high molecular weight and polar glycosyl groups, which limit passive diffusion across intestinal epithelial cells (Wang et al., 2016). Additionally, CHS-IVa undergoes extensive first-pass metabolism via cytochrome P450 enzymes (CYP3A4) and is susceptible to efflux by P-glycoprotein transporters, further reducing systemic exposure. In contrast, structurally modified saponins like ginsenoside Rg3 (with reduced sugar moieties) exhibit improved bioavailability (12%–18%) through enhanced membrane permeability and reduced efflux (Song et al., 2021). Interestingly, the plasma concentration–time curve following PO exhibited a double-peak phenomenon, likely due to enterohepatic circulation (Wang et al., 1999). Such enterohepatic recycling is common in saponins with glucuronide conjugates, as seen in saikosaponin D, yet their systemic distribution remains restricted by rapid renal clearance and tissue sequestration (Rong et al., 2020).

Beyond ginsenosides, the pharmacokinetics of other saponins have also been extensively characterized. Research on AS-IV and its primary metabolite, cycloastragenol (CST), employed a highly sensitive LC–MS method. This method can simultaneously quantify AS-IV and CST using only 20 μL of mouse plasma, demonstrating excellent linearity, precision, and accuracy within 1–200 ng/mL (He et al., 2018). Similarly, total saponins from Dioscorea nipponica (TSSN) exhibited distinct pharmacokinetic profiles, with a prolonged Tmax (approximately 12 h) and a half-life exceeding 11 h. These findings highlight the slower absorption and elimination rates of certain saponins, underscoring the variability in their pharmacokinetics (Yu et al., 2015b; Zhang et al., 2015).

Further studies by Shan et al. investigated the pharmacokinetics of PD from *Platycodon grandiflorum*. The absolute oral bioavailability of PD as a single compound was significantly lower than that in platycodon extract (PRE), suggesting that other PRE components may influence PD’s absorption and metabolism. In PRE, PD concentrations increased within 1–4 h, with a subsequent reduction in hydrolysis rate—potentially due to microbial conversion of other saponins into PD (Shan et al., 2015). Additionally, pharmacokinetic studies of saponins from Gouteng-Baitouweng (GB) have focused on representative components such as anemoside B4, A3, and 23-hydroxybetulinic acid (Tian et al., 2018). Anemoside B4 exhibited the highest exposure in portal vein plasma and liver tissues. In contrast, 23-hydroxybetulinic acid underwent extensive first-pass metabolism (75.1% liver clearance), while anemoside B4 and A3 showed low clearance due to inactive functional groups. The high exposure of anemoside B4 may result from gut microbiota or intestinal enzyme deglycosylation (Xue et al., 2024).

Finally, pharmacokinetic studies of PNS reveal exceptionally low oral bioavailability, estimated at only 1.2%. Among the five primary saponin components analyzed, Rb1 and Rd accounted for approximately 60% of systemic exposure, whereas Rg1 and R1 contributed only 0.7%, with Re undetectable in plasma. The primary metabolites of PNS include protopanaxadiol (PPD), protopanaxatriol (PPT), and compound K (CK) (Wei et al., 2018; Fu et al., 2023). Following IV administration, 17 PNS components were detected in rat plasma, compared to only 10 after PO. Rb1, Rd, and PPD-type saponins are considered the main bioactive components post- PO (Guo et al., 2019). Although Rg1 is the most abundant in oral preparations, its absorption is notably poor. Interestingly, despite PNS’s low bioavailability, its significant pharmacological effects may be attributed to unknown circulating bioactive metabolites, which warrant further investigation.

### 4.2 The safety and toxicity of saponins

While natural saponins demonstrate significant pharmacological potential, many of these compounds may cause adverse effects or toxicity under certain conditions. Therefore, thorough safety and toxicity assessments are essential to guarantee their safe clinical application (Table 3).

**TABLE 3:** Pharmacokinetic characteristics and analysis of saponins: results from acute and subchronic toxicity studies.

Saponin	Type of study	Animals	Method	Dose	Main findings/toxicity manifestations	NOAEL (mg/kg)	Recovery condition	Ref.
20(S)-Ginsenoside Rg3	26-week subchronic toxicity	Beagle	PO	0, 7, 20, 60 mg/kg	The 60 mg/kg group showed an increase in relative kidney weight without other clinical signs of toxicity	20	The relative weight of the kidneys returned to normal after a 4-week recovery period	Gao et al. (2020)
CK	Acute toxicity	SD rats, mice	PO	Max 8000 mg/kg, 10,000 mg/kg	No deaths, no obvious signs of toxicity	—	—	Gao et al. (2019)
CK	26-week subchronic toxicity	SD rats	PO	13, 40, 120 mg/kg	At 120 mg/kg, the male group exhibited systemic toxicity, including weakness, reduced activity, hair loss, slowed weight gain, and apparent liver and kidney toxicity	Male: 40 Female: 120	After a 4-week recovery period, no further signs of toxicity, including liver and kidney abnormalities, were observed in the 120 mg/kg group	Gao et al. (2019)
Dioscin	90-day subchronic toxicity	SD rats	PO	75, 150, 300 mg/kg	Male rats: Weight loss, reduced food consumption, altered urine pH, specific gravity, and proteinuria (75 mg/kg group); hemolytic anemia and mild nephrotoxicity (300 mg/kg group). Female rats: Mild nephrotoxicity and hepatotoxicity	Female: 300	Except for male rats in the 300 mg/kg group, which showed mild gastrointestinal distension and hemolytic anemia, other indicator changes did not clearly correlate with dioscin saponins	Xu et al. (2012)
PD	Single oral dose toxicity test	ICR mice	PO	125, 250, 500, 1000, 2000 mg/kg	No deaths, clinical signs, or changes in body weight were observed. Significant organ weight changes included increased absolute and relative uterine weights in female mice at 250 mg/kg and decreased relative brain weight in male mice at the same dose. Necropsy and histopathological examinations revealed no changes related to PD.	2000	—	Lee et al. (2011)
PS	Chronic toxicity	Male SD rats	PO	200 mg/kg	Chronic liver injury, with a significant increase in serum ALT levels, and fluctuating levels of AST and ALP. Changes in 15 biomarkers, all of which are closely related to liver injury	—	—	Wang et al. (2023)
PNS (rPN)	Acute toxicity	Zebrafish larvae	PO	60–100 μg/mL	Death	0.06	—	Wang et al. (2023)
PNS (dPN)	Acute toxicity	Zebrafish larvae	PO	100–200 μg/mL	Death	0.15	—	Wang et al. (2023)
PNS (dPN)	Acute toxicity	Zebrafish larvae	PO	0.5–5 μg/mL	Weight, body length, and number of vertebrae decreased	0.015	—	Song et al. (2019)

One notable compound is CK, a major metabolite of ginseng. Acute toxicity studies show that CK does not produce significant toxic effects at oral doses of 8 mg/kg in rats and 10 mg/kg in mice. In a 26-week repeated-dose toxicity study, male rats receiving 120 mg/kg exhibited transient weakness, reduced activity, and signs of hepatotoxicity and renal toxicity (Gao et al., 2019). These findings support the safe use of ginsenosides in therapeutic settings. Similarly, 20(S)-ginsenoside Rg3, derived from red ginseng, has raised safety concerns as its usage has increased. To evaluate oral toxicity, Gao et al. conducted a 26-week study in beagles, reporting no significant clinical toxicity in body weight or biochemical markers. However, a significant increase in relative kidney weight was observed at 60 mg/kg, potentially linked to mitochondrial ROS accumulation and tubular epithelial cell apoptosis via Bax/Bcl-2 imbalance, though this effect resolved within 4 weeks. The no observed adverse effect level (NOAEL) was identified as 20 mg/kg, suggesting that 20(S)-ginsenoside Rg3 is safe at this dose (Gao et al., 2020).

In addition to ginsenosides, the safety profile of dioscin, a natural steroidal saponin found in medicinal plants, requires further investigation. Xu et al. conducted a 90-day subchronic toxicity study in Sprague-Dawley rats. Female rats showed no overt toxicity at 300 mg/kg/day, while males exhibited mild gastrointestinal bloating, hemolytic anemia signs, and reduced weight gain at the same dose. The hemolytic effects may arise from dioscin’s interaction with erythrocyte membranes, inducing lipid peroxidation and glutathione depletion (Xu et al., 2012). Urine analysis indicated alterations in pH and protein levels at a dose of 75 mg/kg/day. Hematological assessments revealed that male rats receiving 300 mg/kg/day experienced marked reductions in red blood cell count and hematocrit, suggesting a potential for hemolytic anemia (Xu et al., 2012).

Regarding PD’s acute oral toxicity in mice, no deaths, clinical symptoms, or significant weight changes were observed at doses up to 2000 mg/kg. This suggests the LD_50_ and ALD exceed 2000 mg/kg, supporting clinical safety (Lee et al., 2011). However, a significant increase in uterine weight was noted in female mice at a dose of 250 mg/kg, although no histopathological changes were detected (Erhirhie et al., 2018). Additionally, the safety of Scutellaria baicalensis saponins has been investigated, with long-term studies in rats identifying chronic liver injury as a major concern. Wu et al. observed a significant rise in serum alanine transaminase (ALT) levels, alongside fluctuating aspartate transaminase (AST) and alkaline phosphatase (ALP) levels. Mechanistically, these effects correlate with inhibition of hepatic CYP450 enzymes and disruption of bile acid homeostasis, as evidenced by elevated chenodeoxycholic acid levels (Lee et al., 2011). Metabolomic analysis identified 15 liver injury biomarkers, including acetaminophen glucuronide, correlating with administration duration and dosage (Iluz-Freundlich et al., 2020).

Toxicological challenges extend to Sanqi (Panax notoginseng), known for antihypertensive and antithrombotic properties. Wang et al. evaluated raw (rPN) and decoction (dPN) Sanqi extracts in zebrafish embryos. rPN showed higher acute toxicity (LC50: 73.8 μg/mL) than dPN (LC50: 151.0 μg/mL). Even at 0.5–5.0 μg/mL, dPN caused developmental abnormalities, including reduced weight/length and vertebral malformations (Wang et al., 2023; Song et al., 2019). Developmental banormalities may stem from oxidative DNA damage and p53-mediated apoptosis, as observed in zebrafish embryos exposed to saponin-rich extracts (Mancuso, 2024). Similarly, despite their therapeutic benefits, PS have been shown to induce chronic liver injury with prolonged PO. Metabonomics and UPLC-QTOF-MS analysis identified significant alterations in liver function indicators and biomarkers of liver damage, highlighting the need for further safety evaluations of PNS (Li et al., 2022b; Song et al., 2019).

In summary, while saponins show therapeutic promise, their safety profiles remain inadequately studied. Key concerns include acute/chronic toxicity, hepatotoxicity, and reproductive toxicity. Comprehensive and systematic investigations are urgently needed to establish a robust foundation for safe clinical use of saponins.

## 5 Prospects and challenges

### 5.1 Challenges and strategies for improving the bioavailability of saponin compounds

Saponins exhibit significant potential in modulating macrophage polarization through dual mechanisms of disease prevention and therapeutic intervention. As exemplified by dietary sources such as soybeans, chickpeas, and spinach, naturally occurring saponins function as preventive agents through chronic low-dose exposure. These food-derived saponins lower cholesterol through bile acid binding and micelle disruption, potentially mitigating chronic inflammation linked to atherosclerosis (Xiao et al., 2025). The structural diversity of dietary saponins, particularly their triterpenoid aglycones conjugated with hydrophilic sugar moieties, enables broad-spectrum interaction with lipid membranes and inflammatory pathways. Conversely, therapeutic applications are exemplified by ginsenoside Rh2, which targets colorectal carcinoma through p53-mediated apoptosis and paraptosis. This specific saponin derivative exhibits acute pharmacological actions, including ROS-mediated NF-κB pathway modulation and Bcl-2 family protein regulation, achieving therapeutic efficacy at micromolar concentrations (Li et al., 2020). The preventive-therapeutic duality arises from concentration-dependent bioactivity: dietary saponins maintain immune homeostasis via gradual metabolic modulation, while purified derivatives like Rh2 act through precise molecular targeting. This paradigm highlights the need for differentiated application strategies: food matrix-embedded saponins for long-term disease prevention versus isolated saponin compounds for targeted therapy, with particular promise in cancer immunotherapy through macrophage polarization control.

Meanwhile, the translational potential of this immunomodulatory mechanism is strongly supported by clinical evidence. In a phase II trial, the anti-CSF-1R antibody emactuzumab reprogrammed TAMs from immunosuppressive M2 to antitumor M1 phenotypes in advanced solid tumor patients, correlating with prolonged progression-free survival (Ries et al., 2014). Similarly, RA patients treated with TNF-α inhibitors exhibited reduced synovial M1 macrophage infiltration and increased M2-like reparative phenotypes, paralleling clinical remission (Tardito et al., 2019). These findings underscore the translational potential of macrophage-targeted therapies. However, their clinical application is hindered by significant challenges, particularly their low bioavailability, which complicates the establishment of effective therapeutic concentrations. This limitation is partly rooted in their interactions with gut microbiota and host transporters. For example, ginsenoside Rg1 undergoes deglycosylation by bacterial β-glucosidases, converting it into less polar metabolites like Rh1, which exhibit altered membrane permeability and accelerated systemic clearance (Liu et al., 2011). Similarly, CHS-IVa is hydrolyzed by microbial enzymes in the colon, generating aglycones with reduced intestinal absorption efficiency. These microbial transformations not only diminish bioavailability but also create interindividual variability linked to gut microbiota composition (Zhang et al., 2019). To address this, advanced nano-drug delivery systems—including nanoparticles, nanoliposomes, and nanoemulsions—have emerged as promising strategies. These systems enhance membrane permeability, extend drug circulation time, and enable precise targeted delivery. For instance, ginsenoside Rg3 encapsulated in PEG-PLGA nanoparticles (75–90 nm) significantly amplifies apoptosis in glioma cells (Hu et al., 2025). Notably, nanoencapsulation can shield saponins from microbial degradation by limiting direct contact with gut flora, thereby preserving their structural integrity.

Despite their potential, saponins’ inherent toxicity remains a concern. Exosomes, naturally secreted vesicles, offer an innovative solution for delivering bioactive substances, including genetic material and small molecules—while minimizing off-target effects on healthy cells (Butreddy et al., 2021). These vesicles can traverse biological barriers, such as the blood-brain barrier, and evade immune detection. For instance, exosomes loaded with AS-IV have been shown to upregulate miR-214 expression, thereby enhancing endothelial cell function and promoting angiogenesis (Zou et al., 2022). Structural optimization of saponin compounds is another crucial avenue for mitigating toxicity and improving therapeutic efficacy. For instance, dioscin derivatives like 3-O-tethered triazoles and methotrexate conjugates show higher cytotoxicity and solubility with lower IC_50_ values than the parent compound (Li et al., 2007). Modifying sugar moieties in saponins (replacing glucose with arabinose) can reduce susceptibility to microbial β-glucosidases, as demonstrated in modified CK analogs, which show improved stability in simulated intestinal fluid (Singh et al., 2022). Recent advances in computer-aided drug design (CADD) and artificial intelligence (AI) further accelerate the development of saponin derivatives with improved activity and bioavailability. Techniques such as high-throughput screening, molecular docking, and dynamic simulations enable the rational design and prediction of optimized saponin compounds (Hoshyar et al., 2016). While nanocarriers enhance saponin delivery, their success critically depends on formulation-specific properties. Particle size (<200 nm for optimal tumor penetration), surface charge (near-neutral to avoid rapid clearance), and encapsulation efficiency (>80% for therapeutic payloads) are key determinants of *in vivo* performance (Hoshyar et al., 2016). Moreover, long-term safety concerns persist, as certain nanomaterials may induce mitochondrial toxicity or trigger immune responses through NLRP3 inflammasome activation (El-Kady et al., 2023). Scaling up production while maintaining stability and reproducibility remains a hurdle, as evidenced by the <10% success rate of nanoformulations in phase III clinical trials (Ding et al., 2023). These innovations hold immense potential to amplify the therapeutic efficacy of saponins, paving the way for more effective treatments across a wide range of diseases.

### 5.2 Saponin regulation of macrophage polarization: new research directions

Addressing challenges in saponin research necessitates a deeper understanding of their regulatory roles in macrophage polarization. Recent studies indicate that combining saponins with other bioactive compounds can yield synergistic therapeutic effects. For instance, Khan et al. (2019) demonstrated that a combination of Ganoderma lucidum polysaccharides (GLP) and Gynostemma pentaphyllum saponins (GpS) shifted gut macrophages from the pro-inflammatory M1 phenotype to the anti-inflammatory M2 phenotype in a CRC model (Khan et al., 2019). This shift was associated with decreased pro-inflammatory cytokines (IL-1β, TNF-α) and increased anti-inflammatory cytokines (IL-10, IL-4), along with suppression of tumor-promoting pathways and improved intestinal barrier integrity (Khan et al., 2017). However, emerging evidence suggests that macrophage polarization extends beyond the classical M1/M2 dichotomy. Inflammatory microenvironments can induce mixed or transitional phenotypes, such as M2-like TAMs expressing both CD86 (M1 marker) and CD206 (M2 marker), which exhibit immunosuppressive and pro-angiogenic functions distinct from canonical subsets (Boutilier and Elsawa, 2021). Similarly, metabolic reprogramming (e.g., fatty acid oxidation-driven OXPHOS) in macrophages generates intermediate states (M(Hb) or M4-like phenotypes) that contribute to plaque stability in atherosclerosis or fibrosis resolution (Kelly and O'Neill, 2015). These findings highlight the need to characterize context-dependent macrophage heterogeneity when evaluating saponin-mediated immunomodulation.

Recent advancements in single-cell RNA sequencing (scRNA-seq) have provided critical insights. Yang et al. identified ​four distinct TAM subsets and their influence on the TME (Yang et al., 2021). Meanwhile, Yan et al. combined scRNA-seq with spatial transcriptomics to map glioma subcluster patterns and M2 TAM interactions across tumor regions, revealing cytokine and growth factor dynamics in inflammation and fibrosis (Yan et al., 2024). These studies underscore the therapeutic potential of modulating macrophage polarization, especially in diseases with hyperactive immune responses. For example, Wang et al. showed that luteolin mitigates cytokine storms by regulating bone marrow-derived macrophage (BMDM) polarization, reducing tissue damage. Both luteolin and saponins may share anti-inflammatory mechanisms, offering innovative strategies to control cytokine storms (Wang et al., 2021b).

In addition to acute responses, macrophage polarization is critical in managing chronic conditions such as age-related macular degeneration (AMD), where a balance between destructive inflammatory and reparative states is essential (Cao et al., 2011). Saponins may promote a reparative macrophage phenotype, representing a promising therapeutic avenue for AMD patients. Emerging gene-editing technologies further expand therapeutic possibilities. Zhao et al. used CRISPR-Cas9 with nanovesicles to reprogram TAMs into antitumor M1 phenotypes, significantly suppressing tumor growth and enhancing immune activation in the TME (Zhao et al., 2024). These advancements not only hold promises for cancer treatment but also pave the way for precise therapeutic strategies targeting genetic disorders, autoimmune diseases, and viral infections.

**FIGURE 5:**
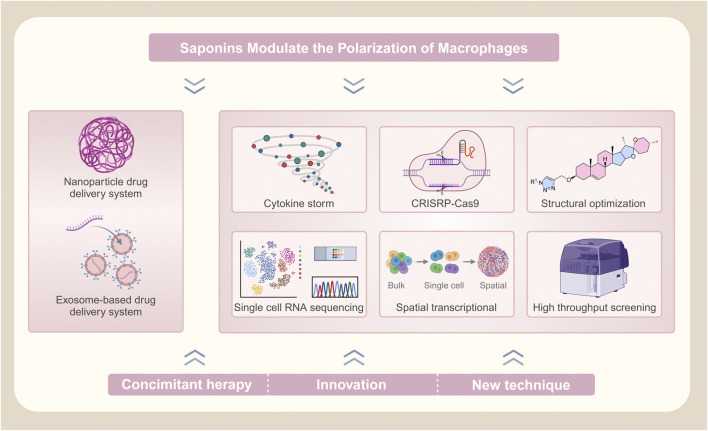
Advancing saponin-mediated regulation of macrophage polarization: challenges and future directions.

In short, advancing our understanding of macrophage polarization and optimizing saponins could enhance their therapeutic applications. Future research should focus on translating insights into clinical practice, exploring saponin combinations with other natural compounds, and leveraging gene-editing technologies to maximize therapeutic potential (Abu-Odah et al., 2022) (Figure 5).

## 6 Conclusion

In conclusion, this review emphasizes the critical roles of saponin compounds in regulating macrophage polarization and their implications for disease progression and therapy. By evaluating their pharmacokinetics and addressing safety concerns, we provide a framework for developing saponins as therapeutic agents. As a diverse class of natural products with significant pharmacological potential, saponins are poised to drive innovation in disease treatment. With advancements in experimental methodologies and technologies, these compounds may lead to novel therapeutic strategies, facilitating breakthroughs in managing various diseases and advancing modern medical science.

## Glossary

5-LOX: 5-lipoxygenase
A3: Anemoside A3
AGEs: advanced glycation end products
ALK5: activin receptor-like kinase 5
ALT: alanine aminotransferase
Arg1: arginase-1
AsC: Araloside C
ASD: Akebia saponin D
AS-IV: astragaloside IV
AST: aspartate aminotransferase
BMDMs: bone marrow-derived macrophages
CADD: computer-aided drug design
CK: ginsenoside CK
COX-2: cyclooxygenase-2
CPA1: carboxypeptidase A1
CRC: colorectal cancer
CS: Chikusetsu saponin IVa
CST: cycloastragenol
Cx43: connexin 43
CYP 4A: cytochrome P450 4A
DSS: dextran sulfate sodium
EAE: experimental autoimmune encephalomyelitis
EMT: epithelial-mesenchymal transition
EsA: Esculentoside A
FIZZ1: inflammatory zone 1
GA: gouty arthritis
HFD: high-fat diet
HIF-1α: hypoxia-inducible factor-1α
HMGB1: high mobility group box 1
IFN-γ: interferon-gamma
IG: oral gavage
IL-10: interleukin-10
IL-1β: interleukin-1β
IL-4: interleukin-4
IL-6: interleukin-6
iNOS: inducible nitric oxide synthase
IRF: interferon regulatory factor
IV: intravenous injection
IκB: κB inhibitors
LPS: lipopolysaccharide
MAPK: mitogen-activated protein kinase
mPGES-1: microsomal prostaglandin E synthase-1
MR: mannose receptor
Mrc1: mannose receptor C type 1
MSU: monosodium urate
mTORC1: mammalian target of rapamycin complex 1
MYD88: Myeloid differentiation primary response 88
NAFLD: non-alcoholic fatty liver disease
NF-κB: nuclear factor κB
NICD: Notch intracellular domain
NOAEL: No Observed Adverse Effect Level
OP: osteoporosis
PD: platycodin D
PI3K: Phosphoinositol-3 kinase
PI3Kγ: phosphoinositide-3 kinase gamma
PNS: Panax notoginseng saponin
PO: oral administration
PPAR-γ: peroxisome proliferator-activated receptor-γ
PS: Pulsatilla saponins;
P-SMAD2: phosphorylated SMAD2
ROS: reactive oxygen species
scRNA-seq: single-cell RNA sequencing
SD: Sprague Dawley
SIRPα: signal-regulated protein α
SOCS: suppressor of cytokine signaling
SPF: specific pathogen-free
SQ: subcutaneous injections
TAMs: tumor-associated macrophages
TGF-β: transforming growth factor-beta
TGFβR1: factor β receptor type 1
TGFβR1: TGFβ receptor 1
TLR4: Toll-like receptor 4
TLRs: Toll-like receptors
TME: tumor microenvironment
TNF-α: tumor necrosis factor-alpha
TSAIII: Timosaponin AIII
VEGF: vascular endothelial growth factor
